# Mountain biodiversity and ecosystem functions: interplay between geology and contemporary environments

**DOI:** 10.1038/s41396-019-0574-x

**Published:** 2020-01-02

**Authors:** Ang Hu, Jianjun Wang, Hang Sun, Bin Niu, Guicai Si, Jian Wang, Chih-Fu Yeh, Xinxin Zhu, Xiancai Lu, Jizhong Zhou, Yongping Yang, Minglei Ren, Yilun Hu, Hailiang Dong, Gengxin Zhang

**Affiliations:** 1 Key Laboratory of Alpine Ecology and Biodiversity, Institute of Tibetan Plateau Research, Chinese Academy of Sciences, Beijing, 100101 China; 2 College of Resources and Environment, Hunan Agricultural University, Changsha, 410128 China; 3 State Key Laboratory of Lake Science and Environment, Nanjing Institute of Geography and Limnology, Chinese Academy of Sciences, Nanjing, 210008 China; 4 Kunming Institute of Botany, Chinese Academy of Sciences, Kunming, 650204 China; 5 Institute of Geology and Geophysics, Chinese Academy of Sciences, Lanzhou, 730000 China; 6 Land and Resource College, China West Normal University, Nanchong, 637009 China; 7 Life Sciences College, Xinyang Normal University, Xinyang, 464000 China; 8 China State Key Laboratory for Mineral Deposits Research, School of Earth Sciences and Engineering, Nanjing University, Nanjing, 210093 China; 9 Department of Microbiology and Plant Biology, Institute for Environmental Genomics, University of Oklahoma, Norman, OK 73019 USA; 10 State Key Joint Laboratory of Environment Simulation and Pollution Control, School of Environment, Tsinghua University, Beijing, 100084 China; 11 Earth Science Division, Lawrence Berkeley National Laboratory, Berkeley, CA 94270 USA; 12 Department of Geology and Environmental Earth Science, Miami University, Oxford, OH 45056 USA; 13 State Key Laboratory of Biogeology and Environmental Geology, China University of Geosciences, Beijing, 100083 China

**Keywords:** Microbial ecology, Biodiversity, Biogeography

## Abstract

Although biodiversity and ecosystem functions are strongly shaped by contemporary environments, such as climate and local biotic and abiotic attributes, relatively little is known about how they depend on long-term geological processes. Here, along a 3000-m elevational gradient with tectonic faults on the Tibetan Plateau (that is, Galongla Mountain in Medog County, China), we study the joint effects of geological and contemporary environments on biological communities, such as the diversity and community composition of plants and soil bacteria, and ecosystem functions. We find that these biological communities and ecosystem functions generally show consistent elevational breakpoints at 2000–2800 m, which coincide with Indus-Yalu suture zone fault and are similar to the elevational breakpoints of soil bacteria on another mountain range 1000 km away. Mean annual temperature, soil pH and moisture are the primary contemporary determinants of biodiversity and ecosystem functions, which support previous findings. However, compared with the models excluding geological processes, inclusion of geological effects, such as parent rock and weathering, increases 67.9 and 35.9% of the explained variations in plant and bacterial communities, respectively. Such inclusion increases 27.6% of the explained variations in ecosystem functions. The geological processes thus provide additional links to ecosystem properties, which are prominent but show divergent effects on biodiversity and ecosystem functions: parent rock and weathering exert considerable direct effects on biodiversity, whereas indirectly influence ecosystem functions via interactions with biodiversity and contemporary environments. Thus, the integration of geological processes with environmental gradients could enhance our understanding of biodiversity and, ultimately, ecosystem functioning across different climatic zones.

## Introduction

It has long been the core of ecology for disentangling the mechanisms underlying temporospatial distributions of biodiversity, and further ecosystem functions [1–4]. Biodiversity is by no means the only, or even the primary, driver of ecosystem functions [5, 6]. Both biodiversity and ecosystem functions have been known to be driven by common drivers of contemporary environments, such as climate and biotic and abiotic attributes [2, 4, 7, 8]. Biodiversity could be also shaped by long-term drivers, such as geological processes, which impart lasting legacies on contemporary environments ([1, 9, 10]). This is especially true for mountain regions, where a close link between geological processes and biological communities has been recently revealed by showing the effect of erosion and soil heterogeneity on biodiversity of terrestrial tetrapods [11]. However, geological processes have not yet been assessed for effects on microbial communities and ecosystem functions. Furthermore, the interactions between contemporary and geological processes have rarely been documented in regard to their influence on biological communities and ecosystem functions.

**Fig. 1:**
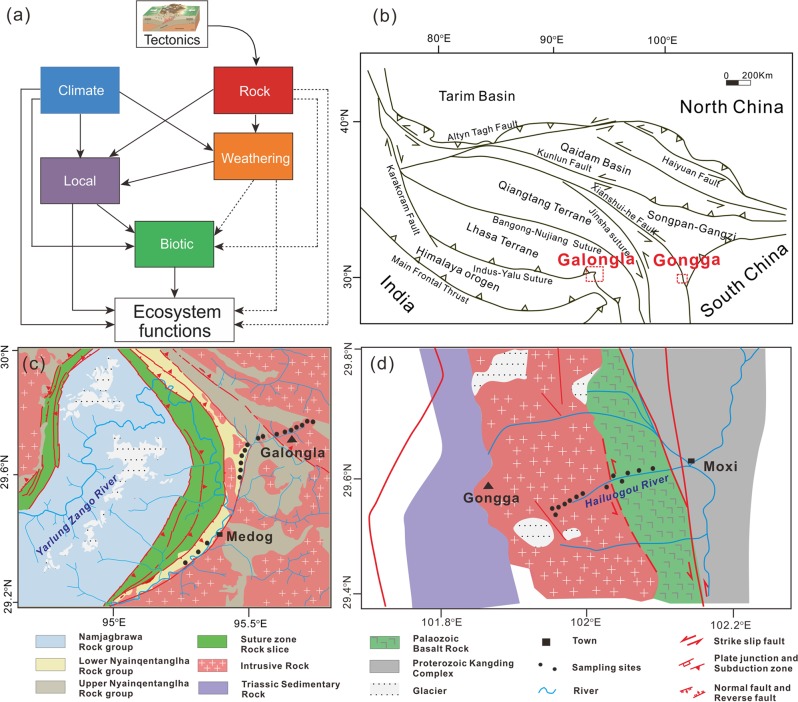
Hypothesized mechanisms for biological communities and ecosystem functions, and sampling maps. **a** Conceptual model showing hypothesized relationships among contemporary environments (e.g., climate, local, and biotic), long-term geological (e.g., parent rock and weathering) processes and ecosystem functions. Solid arrows depict known causal relationships, whereas dashed arrows show the hypothesized but not explicitly documented relationships. **b** Tectonic map of the Tibetan Plateau showing the locations of Galongla and Gongga mountains. **c** Sampling sites on Galongla Mountain in Medog, Tibet. This elevational gradient extends across the Indus-Yalu suture zone fault (red lines) bounded by the Himalaya and Gangdese terranes. **d** The sites sampled on Gongga Mountain by Li et al. [18]. The elevational gradient is intercepted by a secondary Xianshuihe fault (red lines).

To better understand the underlying mechanisms, we propose a framework integrating biology and earth sciences by incorporating both contemporary environments and long-term geological processes on mountainsides (Fig. 1a). Mountain ranges, created and affected by geological processes, have tectonic boundaries, such as sutures and faults [12], and show predictable trends in climatic and abiotic environmental factors with elevation, and thus show plant and animal zonation. For example, the Himalayan–Tibetan orogeny was developed by the collision of the Indo-Malaysia and Eastern-Asia tectonic plates [13, 14] and harbors dozens of major faults [12]. The resulting mountain ranges cover almost all major natural ecosystem types on Earth and are important in maintaining biodiversity and ecosystem functions, such as South-East Tibet biodiversity hotspot [15]. Elevation gradients in mountain regions are invaluable as a natural laboratory for the empirical testing of the hypothesized framework (Fig. 1a) for biodiversity patterns [16, 17] and their links to ecosystem functions.

In this study, we addressed the question of how biodiversity and ecosystem functions vary along an elevational gradient, what are the main drivers for such elevational patterns (contemporary environments versus geological processes), and how geological processes directly and indirectly affect biodiversity and ecosystem functions. Along an elevational gradient of 700–3760 m on Galongla Mountain on the South-East Tibetan Plateau, we examined the biological communities and ecosystem functions for 180 plots at 18 sites covering multiple climatic and vegetation zonations from tropical monsoon rainforests to frigid shrub meadows (Fig. 1b, c). The selected elevational gradient crosses multiple faults with contrasting rock formations that result from the movements and collisions of the Himalaya and Gangdese terranes (Fig. 1b). We examined geological variables, such as soil parent rock and weathering conditions, and contemporary environments, such as climate, local, and biotic attributes (Fig. 1a, S1). For biotic attributes, we determined the relative abundance, diversity, and community composition of plants and bacteria. We further measured 38 ecosystem functions, which are categorized into functional groups including soil nutrients, plant biomass, microbial biomass, and carbon cycling and storage (Table S1), and we characterized three facets of ecosystem functioning: individual function, ecosystem multifunctionality (EMF) [6], and the composition of ecosystem functions. EMF is an integrated ecosystem function metric to indicate the simultaneous performance of multiple functions [6]. We finally tested the consistency in biodiversity elevational patterns by comparing our results to the reported bacterial communities of Gongga Mountain [18] on the South-East Tibetan Plateau (Fig. 1d). The framework regarding “Materials, Methods, and Aims” is shown in Fig. S2. We expected that the biodiversity of plants and microbes, and ecosystem functioning would be strongly affected by contemporary environments, such as soil pH and current climates. We further expected the long-term processes (that is, parent rock and weathering) would directly mediate the biodiversity and ecosystem functioning, but also show indirect influences via the interaction with contemporary environments. These hypothesized links for biological communities and ecosystem functions are shown in Fig. 1a.

## Materials and methods

### Site description

We examined a 3000-m elevational gradient (29°28′–29°75′ N, 95°20′–95°71′ E) on Galongla Mountain in Medog County, the lower reach of the Yalu Tsangpo River in South-East Tibetan Plateau, China (Figs. 1b, c). Galongla Mountain is a part of the Gangrigabu Mountain range, and the elevations go through the Indus-Yalu suture zone fault. This region was highly geologically dynamic as being in a strong tectogenetic area [19]. The Indus-Yalu suture is bounded by the Himalaya and Gangdese terranes, which are originally derived from the Indo-Malaysia and Eastern-Asia plates, respectively.

Along this elevational gradient, a full range of vegetation types is distributed. At the lowest elevation (below 1100 m), there is the northernmost and highest-elevation tropical monsoon rain forest zone in the Northern Hemisphere. Toward high elevations, vegetation zones are the following: subtropical evergreen broadleaved forests (1100–2000 m), subtropical evergreen and semi-evergreen broadleaved forests (2000–2500 m), temperate mixed coniferous broadleaved forests (2500–3000 m), frigid-temperate coniferous forests (3000–3700 m), and frigid shrub meadows beyond 3700 m.

### Vegetation survey and soil sampling

In July–August of 2012, we performed vegetation surveys and soil sampling at 18 sites or elevations ranging from 700 to 3760 m across the six vertical vegetation zones. Each site was carefully selected based on soil formation and no glacial, river, and wind impacts were identified. For plant communities, we established ten plots of 10 m × 10 m on each site for trees, and 5 m × 5 m plots for shrubs. The identification level of plants varied from species to family, and we recorded virtual taxa (hereafter species) and the number of individuals of each species for the estimation of density, coverage, and height within each plot. Plant importance values were computed as the average of the relative density, coverage, and height. We categorized plants as trees (that is, fir, hardwood and softwood), shrubs, and herbs. Plant biomass for these plant types was estimated as follows [20–22]: (1) fir biomass = (0.4642 *V* + 47.4990)/100; (2) hardwood biomass = (0.6573 *V*^1.0502)/100; (3) softwood biomass = (2.1529 *V*^0.6085)/100; (4) shrub biomass = (0.0398 * height * 100 − 0.3326) * coverage/25; and (5) herb biomass = (0.0175 * height * 100–0.2888) *  coverage. *V* represents volume, and was calculated as *V* = 100 * height * coverage.

For soil samples, we randomly collected 25 soil cores from the upper 10 cm using a soil auger (*Ф* 5 cm) from each plot, and then mixed them as one composite sample. Totally, there were ten composite soil samples for each site, and 180 samples for the whole elevational gradient. For each composite sample, 500 g of soils was sieved through a 2-mm mesh and then stored at 4 °C for physiochemical and enzyme activity analyses or at −80 °C for organic chemical and molecular analyses.

### Climate and soil physiochemical variables

For each site, mean annual temperature was predicted with local meteorological stations using a linear model with latitude, longitude and elevation as explanatory variables. Mean annual precipitation was obtained based on the datasets from the Climate Hazards group InfraRed Precipitation with Stations (CHIRPS, http://chg.geog.ucsb.edu/data/chirps/) and local meteorological stations in the study area using least-squares polynomial curve-fitting with elevation [23].

We measured soil physiochemical variables, namely, soil moisture, pH, total nitrogen (TN), total phosphorus (TP), total organic carbon (TOC), water-soluble soil organic carbon (WSOC), water-soluble soil organic nitrogen (WSON), NO_3_^−^-N, and NH_4_^+^-N, according to previous literatures [24, 25].

### Geological variables

The mineral components of soil parent rock, including quartz, plagioclase, K-feldspar, amphibole, muscovite, and chlorite, were identified. We used individual minerals to perform the principal component analysis (PCA, Fig. S3a), and the first two axes of PCA accounting for 61.4% of the total variance were used as explanatory variables representing the soil parent rock. Then, we measured metal elements including Ca, Fe, Mg, Al, K, Na, Mn, and Ti. The first two axes of PCA of these metal elements explaining 80.3% of the variance (Fig. S3b) were used to indicate geochemical factors. We further computed several weathering indices, such as the chemical index of alteration (ClA) [26], and the Ti/Fe, Ti/Al, Mg/Al, and Ca/Al ratios. The detailed measurements of mineral components and metal elements were described in Supplementary Information.

### Phospholipid fatty acids (PLFAs), glycerol dialkyl glycerol tetraether (GDGT), and soil enzyme activities

To further estimate microbial composition and biomass, we analyzed PLFAs [27] and GDGTs [28]. The PLFAs were extracted and measured according to previous literature [29], and the identified PLFA peaks were further grouped into fungi, bacteria, actinomycetes, or protozoa according to their origins [27]. The GDGTs, including isoprenoid GDGTs (iGDGTs) and branched GDGTs (bGDGTs), were extracted and estimated as previously described [30]. The iGDGTs and bGDGTs were derived from Archaea and bacteria, respectively [31]. To determine the activity of soil enzymes, we measured β-glucosidase, amylase, invertase, phenol oxidase, and cellulase as previously described [32]. More details on the extraction and measurements of PLFAs, GDGTs, and soil enzymes could be found in Supplementary Information.

### Bacterial communities

DNA was extracted based on 0.5 g of frozen soils using the FastDNA^®^ SPIN Kit for Soil (MP Biomedicals, USA) following the manufacturer’s instructions. DNA quality was assessed with a NanoDrop ND-2000c UV–Vis spectrophotometer (Thermo Fisher Scientific, USA). PCR amplification of the 16S rRNA gene hypervariable region V4 was performed with the primers 515F (5′-GTGCCAGCMGCCGCGGTAA-3′) and 806R (5′-GGACTACHVGGGTWTCTAAT-3′) [33]. The 2 × 250 bp paired-end sequencing of the PCR amplicons was conducted on a MiSeq platform (Illumina, USA). Sequencing data were analyzed using an in-house Galaxy software platforms [34] (IEG sequence analysis pipeline, http://zhoulab5.rccc.ou.edu:8080), as described in details in Supplementary Methods and Table S2. To ensure that biodiversity estimates were not biased or confounded by variation in sampling intensity (Fig. S4), the bacterial communities were rarefied at 10,000 sequences for the following analyses. We welcome future studies to support our current findings obtained from rarefaction by applying other alternative approaches, such as the mixture model framework proposed by McMurdie and Holmes [35].

### Estimating biological communities

We considered the plant and bacterial communities from three facets: relative abundance, diversity, and community composition. For plants, the relative abundance was calculated for different plant types (that is, tree, shrub, and herb) and each species present in 5–95% of the samples. Species richness was estimated using the number of plant species for all plants and different plant types. Detrended correspondence analysis (DCA) [36] with default 26 segments (the same for the following analyses) was used to evaluate changes in the plant community across different vegetation zones or elevations. The plant community compositions were represented by the first two DCA axes for all plants and the different plant types. The analyses of species richness and DCA were performed using the R package vegan V2.4.6 [37].

For bacteria, we considered the relative abundance at the phylum level. OTUs were agglomerated at the phylum level and the relative abundance was calculated. Species richness was calculated using the number of OTUs for the whole bacterial community and their phyla. We also estimated the alternative metrics, such as Chao 1 [38] and phylogenetic diversity [39] and found they showed strong correlations with species richness (*R*^2^ = 0.97 and 0.96, respectively). Thus, we used species richness in the following analyses. To represent the bacterial community compositions, we used the first two axes of DCA for the whole bacterial community and their phyla by using the total OTU table and the OTU table of each bacterial phylum, respectively. We selected 18 dominant bacterial phyla that were present in more than 80% of the samples by following previous references [40], and such additional analyses were used to support the findings obtained at the whole community level. We divided the *Proteobacteria* phylum into different classes (hereafter phyla) because of the high relative abundance and diversity of *Proteobacteria* and the different ecological functions of these classes.

To summarize biodiversity, we computed a single-index multidiversity (MD) as a synthetic measure [41] by using an approach of averaging standardized values of the species richness of plants and the whole bacterial community, and also the species richness of dominant bacterial phyla. We considered both the whole community and phylum levels because that bacterial phyla have greater phylogenetic or physiological diversity than those of animals and plants [42, 43], and could show distinct biogeographical patterns in biodiversity [40, 44].

### Estimating ecosystem functions

Similar to plant and bacterial communities, we considered ecosystem functions from three facets, that is individual function, EMF, and the composition of ecosystem functions. We quantified 38 ecosystem functions categorized by five functional groups (Table S1): Plant biomass, microbial biomass, enzyme activities, the abundance of photosynthetic bacteria, and soil nutrients. The composition combination of these groups of ecosystem functions was referred as “composition of ecosystem functions”. We included a large number of ecosystem variables to approximate as wide a range of different ecosystem functions as possible [45]. For plant biomass, we considered the biomass, the individual number, the mean height, and the coverage of trees (firs, hardwoods, and softwoods), shrubs, or herbs. For microbial biomass, we examined PLFA concentrations of the groups of bacteria, fungi, actinomycete, and protozoa, and the concentrations of bGDGT, iGDGT, GDGT-0, and Crenarchaeol. For photosynthetic bacteria, we included the relative abundances of *Cyanobacteria*, *Rhodospirillales*, *Rhodocyclales*, and *Chlorobi*. Finally, for soil nutrients, we considered the concentrations of TOC, TN, TP, WSOC, WSON, NH_4_^+^-N, and NO_3_^−^-N. Although we have included 38 ecosystem functions, some important functions are inevitably unmeasured, such as plant available phosphorus, carbon fixation, and nitrogen fixation, and future studies are encouraged to include more essential functions for comprehensive understanding of ecosystem functioning.

For EMF, we used an averaging approach, which aims to collapse multifunctionality into a single metric that estimates the average value of multiple functions observed in a given sample [2]. We calculated *Z*-scores for all variables evaluated, and the EMF was the average *Z*-score for all functions measured for each sample. EMF's for all functions and the above five functional groups were estimated. In addition, in order to evaluate the effects of the number of ecosystem functions on EMF, we calculated EMF using a series of all possible combinations from 10 to 38 functions with 1000 permutations. These EMF analyses were performed by using the R package multifunc V0.8.0 [46].

For the composition of ecosystem functions, we used the first two axis scores of DCA to assess the changes in the compositions of all ecosystem functions and also specific functional components, such as PLFAs, GDGTs, and enzyme activities, across the vegetation zones or elevations.

### Statistical analyses

We used the following variables, including those related to contemporary environments and long-term geological processes, as explanatory variables. Contemporary processes included climate (i.e., temperature and precipitation), local (i.e., soil pH and moisture), and biotic attributes (i.e., the diversity and DCA of plants and bacteria). Geological variables included soil parent rock (i.e., quartz, plagioclase, K-feldspar, amphibole, muscovite, chlorite, and the first two PCA axes of minerals) and weathering conditions (i.e., CIA, Ti/Fe, Ti/Al, Mg/Al, and Ca/Al ratios, and the first two PCA axes of metal elements). Detailed information about all explanatory variables is listed in Table S3. The response variables included biological communities (plants and bacteria) and ecosystem functions at the three abovementioned facets, and were analyzed for their elevational patterns and underlying drivers.

#### (1) Elevational breakpoints of plants, bacteria, and ecosystem functions

The relationships between elevations and explanatory or response variables were visualized with loess regression models. We further tested the breakpoints or abrupt changes for all explanatory and response variables along the elevational gradient using a piecewise linear regression analysis [47, 48] with the R package SiZer V0.1.5. We calculated bootstrapped confidence intervals for the breakpoint estimates [48]. The sampling sites along the elevational gradient on Galongla Mountain extend across the Indus-Yalu suture zone fault, which is located at 2293–2438 m (Fig. 1c). Therefore, we defined the elevational band for potential breakpoints within the range of 1800–3000 m in piecewise linear regressions. Furthermore, these breakpoint estimations of the compositions of plants, bacteria, and ecosystem functions were supported or explained by the following statistical analyses.

First, we visualized the separation among sites or elevations by the gradient of fitted contours, which implies a linear relationship between elevations and DCA ordinations of the biological communities and ecosystem functions. We further evaluated the significance of the compositional differences among the elevational zones by the permutational multivariate analysis of variance (PERMANOVA) method with pseudo-F statistic [49]. Heterogeneity of multivariate dispersion was not tested because PERMANOVA could allow rigorous meaningful analysis of even those having variables with extremely non-normal or overdispersed behavior [50]. This analysis was performed using the R package vegan V2.4.6 [37].

Second, we assessed the compositional changes and identified the important breakpoints across multiple species along the elevational gradient with gradient forest analysis [51]. In addition, we also estimated the standardized density of splits for bacteria across the taxonomic levels from genus to phylum where we agglomerated OTUs and thus the whole communities were considered at coarser taxonomic levels than species. These analyses were performed using R packages gradientForest V0.1.17 [51] and extendedForest V1.6.1 [52].

Third, we examined the Bray–Curtis dissimilarity [53] of biological communities or ecosystem functions between pairs of adjacent elevations to identify abrupt elevational compositional changes. The significance and magnitude of dissimilarity differences between adjacent sites were evaluated with PERMANOVA method with pseudo-F statistic. Within the elevation range of 1800–3000 m, we considered the elevational breakpoint as the highest compositional turnover between adjacent elevations, which is defined as the highest pseudo-F statistic and dissimilarity value.

Fourth, we tested the regional consistency in elevational breakpoints by further examining bacterial communities from Gongga Mountain. This mountain is located on the South-East Tibetan Plateau and is over 1000 km away from Galongla Mountain. These two mountains have similar latitudes at 29–30°N and the sampling sites shared the elevation ranges from 1800 to 3800 m. Gongga Mountain is a part of Hengduan Mountain range, and the elevations go through a secondary Xianshui-he fault with contrasting origins of rock formations (Fig. 1c). We collected the bacterial 16S rRNA sequences (NCBI Accession number PRJEB15866) from Li et al. [18], and processed the dataset, along with our data with the bioinformatic methods as described above. We examined the community variations between the two mountains from the following three perspectives: (1) We visualized the bacterial community compositions of the two mountains by DCA ordination with the elevational gradient of fitted contours. (2) We examined pairwise Bray–Curtis similarities between the bacterial communities at similar elevations of the two mountains to test the abrupt compositional changes along the elevational gradients. (3) We calculated the community Bray–Curtis similarities between each elevation of Gongga Mountain and all lower (or upper) elevations of Galongla Mountain and then examined the elevational patterns of these similarities. The upper and lower elevations were defined according to the elevational breakpoint identified from the piecewise linear regression analysis on the whole community compositions of plants or bacteria. The relationships between the community similarity and elevation were fitted and tested with a linear model and permutation tests in the R package lmPerm V. 2.1.0 [54].

#### (2) Drivers of biological communities and ecosystem functions

To evaluate the role or the relative importance of contemporary environments and long-term geological processes on biological communities or ecosystem functions, we performed various statistical methods, such as multimodel averaging [55], gradient forest [51], multiple regression, structural equation models (SEM) [56], variation partitioning (VPA) [57], and random forest analyses [58] (Fig. S2). In particular, the latter four analyses provided knowledge on whether the geological processes in the models provide significantly additional predictive strength for biological communities or ecosystem functions compared with those models with contemporary environments only. The analyses of multimodel averaging and SEM are described as below, while the others are detailed in Supplementary Information.

First, the multi-model approach could provide a quantitative measure of the relative importance of each explanatory variable [55, 59]. Before doing the analyses, strongly correlated variables were dereplicated according to their correlation. Statistical dependence between the explanatory variables was assessed using Pearson correlation coefficients (two-tailed). One of the two variables was selected if their Pearson correlation is higher than 0.7. For plants and bacteria, we used the diversity and compositions of the whole plants and bacteria, and the relative abundance of species as response variables. For ecosystem functions, we used individual function, EMF and the composition of ecosystem functions as response variables. All variables were *Z*-score transformed to estimate the conditional model-averaged parameter as standardized beta values, which enable us to compare the influences of different explanatory variables with different measurement units on biological communities and ecosystem functions.

We further explored the relationships of EMF and explanatory variables with the increasing number of functions, using multimodel averaging and Pearson correlation analyses. The relative influences of explanatory variables were quantified for climate, parent rock, weathering, local, and biotic variables, which was done by selecting variables with the highest absolute standardized beta values. The multi-model averaging analysis was performed by using the R package MuMIn V1.40.4 [60].

Second, SEM was used to test and quantify the interactive effects of contemporary environments and geological variables on biodiversity MD or ecosystem functions EMF. The approach begins by hypothesizing the underlying structure of causal pathways as shown in Fig. 1a. Then, the model is translated into regression equations, and these equations are evaluated against the data to support or refute the hypothesized paths. Through this process, SEM provides an understanding of direct and indirect effects of contemporary and geological processes on MD or EMF. To estimate the predictive strength of geological processes, we fit separate models for predictor variables including and excluding geological processes. ANOVA was used to test the significance of these two separate models. Before modeling, all variables in the SEMs were *Z*-score transformed to allow comparisons among multiple predictors and models. Similar to previous studies [61], we used composite variables to account for the collective effects of climate, parent rock, weathering, local, and biotic attributes, and the candidate observed indicators were shown in Table S3. The indicators for each composite were selected based on the multiple regressions of MD or EMF and the formula for calculating the composite variable for SEM models of MD and EMF were shown in Table S4. Based on all the hypothesized paths among composite variables (that is, full model; Fig. 1a), we examined all alternative models using AIC and overall model fit statistics [62]. We chose the final model that met the model fit statistics with the lowest AIC value. The detailed modeling fit indices for all alternative models were provided in Table S5. Adequate model fits were determined according to a non-significant *χ*^2^ test (*P* *>* 0.05), low AIC value, high comparative fit index (CFI > 0.95) and low standardized root mean squared residual (SRMR < 0.05). We implemented SEMs using the R package lavaan V.0.5.23 [63], which provides multiple-latent variable models by utilizing path diagrams to explain the underlying relationships in the models.

## Results and discussion

**Fig. 2:**
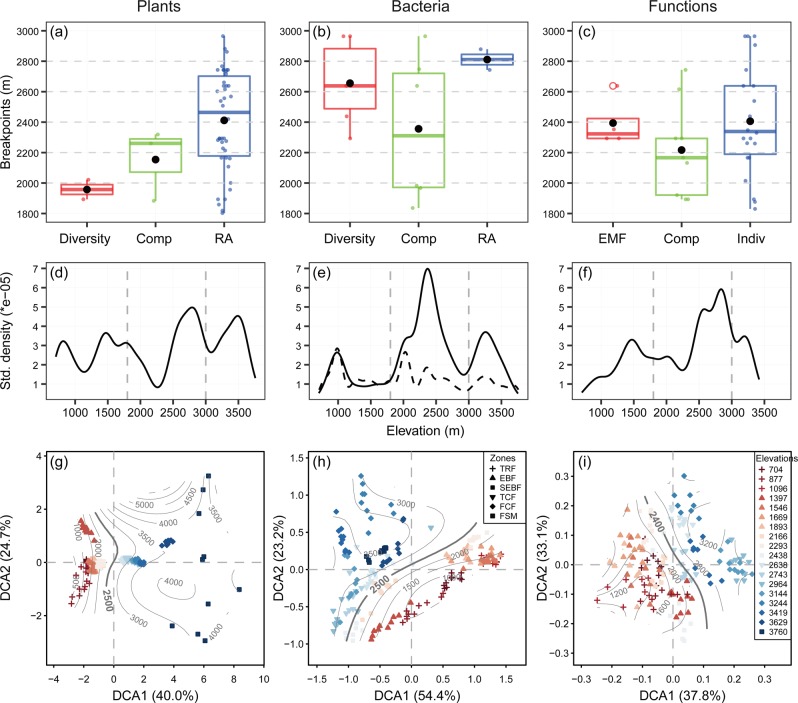
Elevational patterns of plants, bacteria, and ecosystem functions. **a**–**c** The boxplots of elevational breakpoints (i.e., elevations with abrupt changes, colored dots) revealed by piecewise regression analyses [47, 48]. We considered three facets of plants: diversity and community composition (Comp) of whole plants and their types, and relative abundance (RA) of plant types and species. For bacteria, three facets are: diversity and community composition of whole bacteria and their phyla, and relative abundance of bacterial phyla. For ecosystem functions, three facets are: ecosystem multifunctionality (EMF) and composition of all ecosystem functions and their functional groups, and individual functions (Indiv). The average value of breakpoint elevations for each facet is shown with black point. The standardized density of splits showing where important changes in the abundance of multiple species or functions occur along the elevational gradient and indicating the compositional turnover. The standardized density of splits was determined by gradient forest analyses [51] for the species level of plants (**d**) and bacteria (dashed line, **e**), the class level of bacteria (solid line, **e**), or 38 ecosystem functions (**f**). The vertical dashed lines show the 1800–3000 m elevation ranges. One main peak within 1800–3000 m elevation ranges was more obvious at class than species level of bacteria. This is because broader taxonomic classification may balance the distribution uncertainty associated with finer taxonomic resolution and strengthen the phylogenetic conservatism [80]. Detrended correspondence analysis (DCA) plots of the composition of plants, bacteria, and ecosystem functions. DCA analyses were based on the species level of plants (**g**) and bacteria (**h**), and 38 ecosystem functions (**i**). The contours in gray indicate linear relationships between DCA ordination values and elevations. The compositional differences among pairwise elevations were analyzed to determine the highest compositional turnover as shown with bold lines (Fig. S5). Vegetation zones comprise tropical monsoon rain forest (TRF), subtropical evergreen broadleaved forest (EBF), subtropical evergreen and semi-evergreen broadleaved forest (SEBF), temperate mixed coniferous broadleaved forest (TCF), frigid-temperate coniferous forest (FCF), and frigid shrub meadows (FSM).

Generally, for the elevational gradients of biological communities and ecosystem functions, there were significant (*P* *<* 0.05) elevational breakpoints that mostly occurred at 2000–2800 m according to piecewise regression analyses [47, 48] (Fig. 2a–c, Table S6). In particular, the elevational variations for plant (Fig. 2g, S5a) and bacterial composition (Fig. 2h, S5b) showed significant breakpoints at ~2400–2500 m. Both taxonomic groups showed the highest compositional turnover around these breakpoints, which was quantified by pairwise Bray–Curtis dissimilarity (Fig. S6a, b) and gradient forest analyses [51] (Fig. 2d, e). This finding is supported by bacterial compositional turnover from the phylum to genus levels (Fig. S7a) and is more obvious toward higher taxonomic levels, such as class and order levels (Fig. 2e, S7a). The EMF of all 38 ecosystem functions, which is not likely to be affected by the number of functions considered (Fig. S8), had a breakpoint at 2293 m (Fig. 2c). The EMF for the functional groups, such as plant and microbial biomass and soil nutrients, showed significant breakpoints at 2200–2400 m (Fig. 2c, S9). Similar patterns were observed for elevational changes in the composition of ecosystem functions (Figs. 2c, 2i, S5c) and the highest compositional turnover occurred at 2600–2800 m (Fig. 2f, S6c). These breakpoints were particularly apparent for the compositions of some functional groups, such as phospholipid fatty acids [27], glycerol dialkyl glycerol tetraether [28], and extracellular enzymes essential for carbon cycling and storage, based on elevational variations in their compositions (Fig. S10, S5d–f) and the highest compositional turnover within the 1800–3000 m range (Fig. S6d–f).

These consistent elevational breakpoints in the community compositions of multiple taxonomic groups and ecosystem functions have less been reported in previous studies. The breakpoint elevations of bacterial communities are, however, unexpectedly consistent with those at 2600–2800 m for the diversity [18] and community composition (Fig. S11) of soil bacteria on Gongga Mountain (Fig. 1d), which is located over 1000 km east of the studied Galongla Mountain. Furthermore, the bacterial community similarity between pairwise sites with similar elevations of the two mountains was lowest at the breakpoint elevation of 2360 m (Fig. S11b). It should be noted that these two mountains are from two different mountain ranges, Gangrigabu and Hengduan Mountains, which have different orientations and mountain-building origins as a result of collision and extrusion of tectonic plates or terraces (Fig. 1b–d). Such breakpoint elevations are different from the corresponding treelines at 3600–3700 m on the two mountains and contrast with the reported breakpoints of soil bacteria occurring at the treelines on the other mountains [64]. Climate, such as temperature, is widely accepted to be the primary control for the treeline formation and maintenance of biological communities [65]. Our findings however indicate that biological communities and ecosystem functions may not be always dominantly influenced by climate, and further suggest their links to other new processes, such as the legacies of geological events. This argument is supported by two observations. First, the elevational bands that contain breakpoints surprisingly coincide with the locations of faults. Specifically, the Indus-Yalu suture zone fault [66] and the Xianshui-he fault [67] pass through the elevational bands of 2300–2500 m and 2600–2800 m on the Galongla and Gongga Mountains, respectively. Most of chemical characteristics, including minerals, weathering indices, and soil properties, were significantly (*P* < 0.05) different across the Indus-Yalu suture zone fault (results not shown). Second, the fault location on Galongla Mountain was supported by the elevational profile of the geological variables, such as the parent rock and weathering conditions, which showed breakpoints at ~2400 m on average (Fig. S12).

**Fig. 3:**
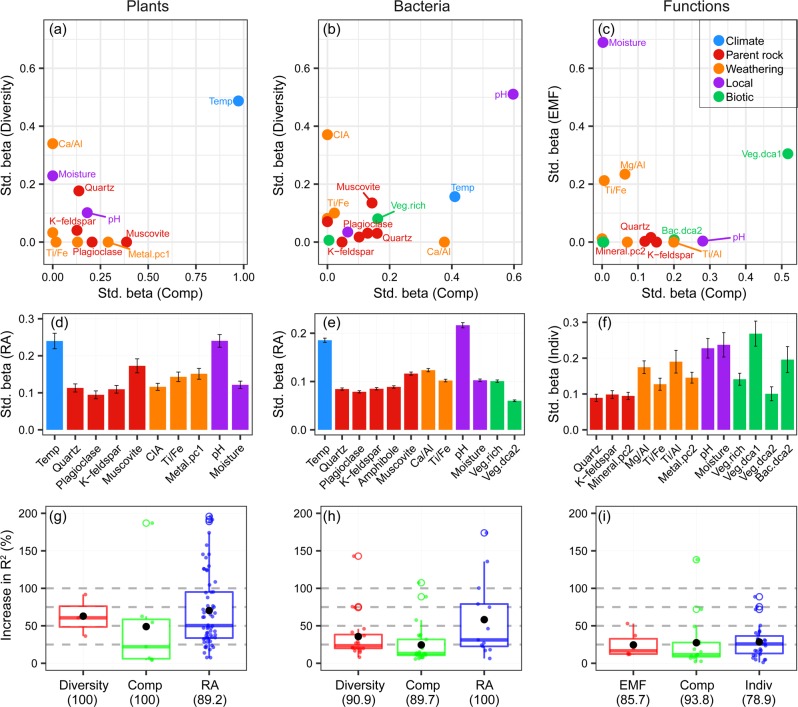
Relative importance of contemporary and geological processes on plants, bacteria and ecosystem functions. First, the relative importance was quantified by the weighted averaging of parameter estimates over best-fitting models [59] by including the five groups of predictors (Fig. 1a): climate, parent rock, weathering, local, and biotic attributes. For plant (**a**, **d**) and bacterial (**b**, **e**) communities, we considered three facets: diversity (**a**, **b**), composition (Comp, **a**, **b**), and relative abundance (RA) of species (**d**, **e**). For ecosystem functions (**c**, **f**), the three facets are: ecosystem multifunctionality (EMF, **c**), the composition of ecosystem functions (**c**) and individual function (Indiv, **f**). Data in **d**–**f** are presented as the means ± s.e. Moreover, compared with the models of contemporary variables, we quantified the improvements in the explained variances by further including geological variables based on stepwise multiple regressions and Akaike’s information criterion [81]. Relative to those in the models of contemporary variables, the improvements in the explained variances by including geological processes were shown as the percent increases in the model *R*^2^ on the three facets of plant (**g**) and bacterial (**h**) communities and ecosystem functions (**i**). The significance in model *R*^2^ increase was determined with ANOVA between the models including and excluding geological variables. The values in parentheses indicate the percentage of significant *R*^2^ increases among all possible models of contemporary variables for each facet. Black points in the boxplots are the mean values for each facet. The response variables were the same variables at three facets of plants, bacteria, and ecosystem functions as mentioned in Fig. 2a–c.

Further statistical analyses to explore the key drivers of biological community and ecosystem functions reveal that both short- and long-term processes showed profound influences, including climate, parent rock, weathering, local, and biotic variables (Fig. 3, S7b, S13–S19). Among contemporary variables, mean annual temperature and soil pH had the strongest effects on the plant and bacterial communities, respectively (Fig. 3a, b, d, e). Ecosystem functions were most strongly affected by soil moisture, followed by plant communities (Fig. 3c). These findings support previous reports showing the important roles of contemporary environments, such as climate and local abiotic variables, in controlling global biodiversity [4, 11, 68–72] and the roles of local abiotic variables and biodiversity in regulating ecosystem functioning [2, 73–75].

Furthermore, geological processes were found to be important as well in not only the plant and bacterial communities, but also ecosystem functions. For instance, bacteria were strongly correlated with weathering indices, that is, the chemical index of alteration [26] and the Ca/Al ratio, when we considered the three facets of bacteria: the diversity and community compositions of total bacteria (Fig. 3b) and their phyla (Fig. S17), and the relative abundance of species (Fig. 3e). These facets of plants were significantly (*P* < 0.05) correlated with the Ca/Al ratio, the geochemical composition, and primary minerals, such as muscovite, plagioclase, and quartz (Fig. 3a, d). EMF and the composition of ecosystem functions were also influenced by plant communities, as well as by weathering indices, such as Mg/Al and Ti/Fe ratios (Fig. 3c, f). Furthermore, for the three facets of plant and bacterial communities and ecosystem functions, we found improvements in the explained variances in the models by including geological processes, of 67.9 and 35.9% on average for plant and bacterial communities, respectively, and of 27.6% on average for ecosystem functions, relative to those in the models excluding geological processes (Fig. 3g–i). Thus, these findings suggest biological communities or ecosystem functions are driven by common mechanisms, that is contemporary environments and the new driver as long-term geological processes.

**Fig. 4:**
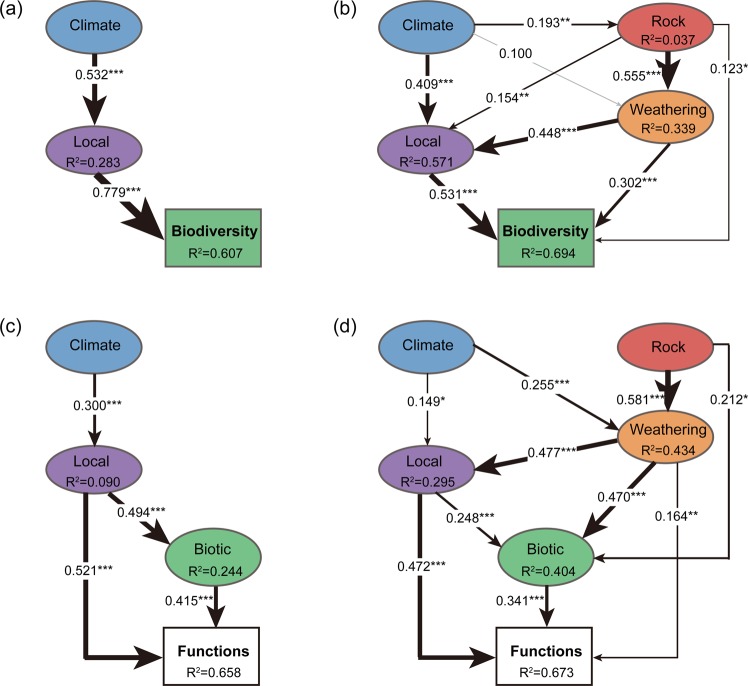
Structural equation models of biodiversity and ecosystem functions. The biodiversity and ecosystem functions are quantified with single-index multidiversity (MD) and ecosystem multifunctionality (EMF), respectively. Best-fitting models illustrate the effects of predictor variables on MD (**a**, **b**) or EMF (**c**, **d**) by excluding (**a**, **c**) or including (**b**, **d**) geological variables. *R*^2^ denotes the proportion of variance explained for endogenous variables. Gray and black arrows indicate statistically non-significant and significant (***P < 0.001, **P < 0.01, *P < 0.05) relationships, respectively. Arrow widths and accompanying numbers are the relative effects (that is, standardized path coefficients) of modeled relationships. Composite and observed variables are indicated in ovals and rectangles, respectively. More details on the model fit are summarized in Table S5.

However, the interactive effect of the contemporary environments and long-term geological processes remains poorly understood. We thus applied SEMs [56] to statistically synthesize their hypothesized relationships (Fig. 1a). To summarize biodiversity, we computed a single-index MD parameter as a synthetic measure [41] by including the species richness of plant and whole bacterial community, and also the species richness of bacterial phyla. When all possible links among these two processes and ecosystem properties were considered (Fig. 1a), geological variables were retained in the final best-fitting SEMs and were revealed to improve the predictive power of the MD and EMF indices (Fig. 4, S20, Tables S5, S7, S8).

Furthermore, our SEMs showed that geological processes had divergent effects on MD and EMF due to the differences in their influence strength and directions. First, geological processes had a greater effect on MD than they did on EMF—after including geological variables, the explained variations of the MD and EMF models were improved by 8.7% and 1.5%, respectively, but only the improvement of the MD model was significant (*P* < 0.001, ANOVA) (Fig. 4a, b). Such divergent effects on MD and EMF are probably because biodiversity is functionally redundant [76, 77], so that the changes in MD do not necessarily alter EMF. Second, geological variables showed direct effects on MD, and mainly indirect influences on EMF via the interactions with biodiversity and contemporary abiotic environments. Specifically, geological factors, such as parent rock compositions, had strong correlations (*R* > 0.55, *P* = 0) with weathering and showed total indirect effects of 0.38 and 0.34 on MD and EMF, respectively (Fig. 4b, d, Table S9). Weathering had significant direct effects on both MD (*R* = 0.30, *P* = 0) and EMF (*R* = 0.16, *P* = 0.008) and a strong indirect effect of 0.43 on EMF via local and biotic variables (*R* > 0.47, *P* = 0; Fig. 4b, d). Such divergent effects of geological processes were further statistically supported by variation partitioning analyses [57] (Fig. S21) and random forest analyses [58] (Fig. S22). For instance, the geological variables alone explained ~10.0% and 1.0% of the variation in MD and EMF, respectively (Fig. S21), and improved the predictive power of MD and EMF by 4.3% and 3.9%, respectively (Fig. S22). Thus, beyond those known from previous studies [2, 3, 11], our findings reveal additional links between geological processes relevant to tectonic dynamics and biological community or ecosystem functions, as previous studies of ecosystem functions have been largely concerned with effects of contemporary biotic and abiotic factors.

Because geological processes related to tectonic plate collision and faults are important on the Tibetan Plateau and in other biodiversity hotspots in mountain regions, our findings have several substantial implications regarding the effects of geological processes on biodiversity and ecosystem functions.

Firstly, our findings highlight the need to integrate data relating to the long-term geological history when making current inferences and future predictions of biodiversity or ecosystem functioning. This study, for the first time, include geological processes to quantify the relative contributions of past geological events on microbial communities and ecosystem functions. Our findings indicate that past geological processes leave a strong signature on the current distributional patterns of biodiversity and overall ecosystem functioning. Specifically, both parent rock and weathering had an indirect influence on ecosystem functions, whereas weathering had a considerable direct effect on biodiversity. Thus, our experimental strategy of explicitly considering geological events could be adopted, as it may facilitate the possibility of identifying and strengthening of the effects of geological processes on the biodiversity and ecosystem functioning.

Secondly, it is possible that elevational breakpoints of biodiversity may be observed in other geological regions with tectonic faults, and that biological communities could serve as indicators of past geological events, such as the formation of terrace boundaries or faults. This is because the consistent elevational breakpoints on the Galongla and Gongga Mountains are largely affected by faults attributed to past geological processes, even though these geological influences are from two different mountain ranges on the South-East Tibetan Plateau. The consistency in breakpoints on the two mountains indicates that the distributional patterns of biodiversity in other regions on the Tibetan Plateau or even around the world might show abrupt changes in similar and predictable ways across faults or even terrace boundaries. However, we should note that this implication of biological indicators was speculated based on two mountains, and current findings need further supports from other mountains, regions and continents.

Finally, further studies are encouraged to study the legacies of past geological events on biological communities and ecosystem functions, involving sampling along multiple elevational transects across faults and considering further more taxonomic groups with fine-resolution analytical approaches. For example, the studies in fungi [4] and animals [11, 78] or in multiple regions with similar geological processes, such as plate collision and tectonic faults, would be required to confirm the generality of our conclusions regarding the new links between long-term geological processes and biodiversity or ecosystem functioning. Furthermore, future studies are encouraged to include other drivers important for observed biogeography of plants and animals, such as seasonality, climate zones and geographical regions, at multiple time points in sampling strategy for comprehensive conclusions. A population ecology approach could be further applied to improve the methodology by evaluating genome legacies from a biological evolutionary perspective [79].

## Supplementary information

Supplemental material

## Data Availability

The 16S rRNA sequences were deposited in NCBI Short Read Archive under accession number PRJNA556333. Other raw data were uploaded to the Dryad repository (10.5061/dryad.fxpnvx0nb). Scripts for statistical analyses are available from the corresponding author upon reasonable request.
