# Supplementary material for: Mountain biodiversity and ecosystem functions: interplay between geology and contemporary environments
Source: ISME J. 2020 Jan 2;14(4):931–44. doi: 10.1038/s41396-019-0574-x (PMC7082341; doi:10.1038/s41396-019-0574-x)
Supplement: Supplementary file 1 — Supplemental material [file 41396_2019_574_MOESM1_ESM.docx]

Supplementary Information for

**Mountain biodiversity and ecosystem functions: interplay between geology and contemporary environments**

Ang Hu^1,2,†^, Jianjun Wang^3,†^, Hang Sun^4^, Bin Niu^1^, Guicai Si^5^, Jian Wang^6^, Chih-Fu Yeh^3^, Xinxin Zhu^4,7^, Xiancai Lu^8^, Jizhong Zhou^9,10,11^, Yongping Yang^4^, Minglei Ren^3^, Yilun Hu^1^, Hailiang Dong^12,13^, Gengxin Zhang^1,#^

**The file includes:**

Supplementary Materials and Methods

Supplementary Tables (S1–S9)

Supplementary Figures (S1–S22)

**Materials and Methods**

***Geological variables***

To identify the mineral components of soil parent rock, we mixed 0.15 g of soil and 0.15 g of corundum and ground them into a powder (~200 mesh) using an agate mortar. The powder was pressed into a disk-shaped wafer. Soil mineralogy was determined by using ARL X’TRA X-ray diffraction (XRD) with CuKα radiation at 40 kV (Thermo Electron, Switzerland). A 2-tetha scan was conducted from 3° to 70°, with an increment of 0.02° per step. Six mineral phases were identified: quartz, plagioclase, K-feldspar, amphibole, muscovite, and chlorite.

As described previously ([Yang *et al.*, 2016](#_ENREF_19)), we performed pressurized acid digestion of soils using a mixture of HNO_3_/HF in a Teflon digestion vessel, and measured metal elements, including Ca, Fe, Mg, Al, K, Na, Ba, Mn and Ti, with Inductively Coupled Plasma-Optical Emission Spectroscopy (Leeman Labs, USA). We used individual metal elements to perform principal component analysis (PCA) based on Euclidean distance. Elements were normalized with respect to Al due to its immobility during weathering. The chemical index of alteration (CIA) was calculated with the formula CIA = [(Al_2_O_3_) / (Al_2_O_3_ + CaO + K_2_O + Na_2_O)] * 100 ([Nesbitt and Young, 1982](#_ENREF_12)).

***Phospholipid fatty acids (PLFAs), glycerol dialkyl glycerol tetraether (GDGT) and soil enzyme activities***

The PLFAs were extracted and measured as previously described ([Frostegård and Bååth, 1996](#_ENREF_4)). Briefly, 5.0 g of freeze-dried soils was extracted with 19 ml of a single-phase mix of chloroform:methanol:citrate buffer solution (1:2:0.8, v/v/v, pH=7.4). After the extraction, the collected non-polar phase was fractioned into three fractions (that is, neutral lipids, glycolipids and phospholipids) by sequential elution with chloroform (6 ml), acetone (6 ml) and methanol (3 ml), respectively, using pre-packed silica solid phase extraction columns Cleanert™ Silica-SPE (Bonna-Agela, USA). The phospholipid fraction was then methylated with a methanol/toluene (1:1) solution (1 ml) and 0.2 M methanolic KOH (1 ml) to produce fatty acid methyl esters. After the addition of fatty acid 19:0 as an internal standard, the samples were analysed on an Agilent 6890A gas chromatograph (Agilent, USA) and identified with a microbial identification system, MIDI Sherlock V4.5 (MIDI, USA). According to the presence and abundance of the signature fatty acids ([Hill *et al.*, 2000](#_ENREF_6), [Si *et al.*, 2014](#_ENREF_15)), the PLFA profiles were assigned to specific organisms or groups of organisms, such as fungi, bacteria, actinomycetes and protozoa.

The GDGTs were extracted and estimated as previously described ([Yang *et al.*, 2014](#_ENREF_18)). Briefly, approximately 6.0 g of freeze-dried soils was ultrasonically extracted with 20 ml dichloromethane (DCM)/methanol (9:1, v/v) for 15 min for 3 times. The obtained total extracts were rotary-evaporated under near-vacuum conditions and separated on an activated Al_2_O_3_ column, using hexane/DCM 9:1 (v/v) and DCM/methanol 1:1 (v/v), into apolar and polar fractions, respectively. The polar fraction, containing the bGDGTs, was then dried under a continuous N_2_ flow, ultrasonically dissolved in a hexane/propanol 99:1 (v/v) mixture at a concentration of 2 mg ml^-1^ and filtered through a 0.2 μm PTFE filter. The identification and quantification of GDGTs were conducted using high-performance liquid chromatography/atmospheric pressure chemical ionization-mass spectrometry (Agilent, USA).

Soil enzymes, including β-glucosidase, amylase, invertase, phenol oxidase and cellulose, were extracted and measured as previously described ([Guan, 1986](#_ENREF_5)). Briefly, β-glucosidase was determined by incubating 1.0 g of soils with 1 ml of 5 mM p-nitrophenyl-β-glucopyranoside for 1 h at 37 °C. Amylase was measured by incubating 5.0 g soils with 10 ml of 1% starch for 24 h at 37 °C. Invertase was measured by incubating 5.0 g soils with 15 ml of 18% sucrose for 24 h at 37 °C. Cellulase activity was determined by incubating 10.0 g soils with 5 ml of 1% carboxymethyl cellulose for 72 h at 37 °C. The suspension of these incubations described above further reacted with 3 ml of 3,5-dinitrosalicylic acid for the colorimetric assay, and the absorbance was read at 508 nm with Microplate Reader (SpectraMax M5, Canada). Phenol oxidase was determined by incubating 1.0 g soils with 10 ml of 1% pyrogallic acid for 2 h at 30 °C, and measured for the colorimetric assay at the absorbance of 430 nm using Microplate Reader (SpectraMax M5, Canada).

***Sequence analyses***

Briefly, all raw sequences were first separated to samples via sample-specific barcodes. The forward and reverse reads of the same sequence were combined with at least a 20 bp overlap and < 5% mismatches using FLASH ([Magoč and Salzberg, 2011](#_ENREF_10)). The unqualified sequences were filtered with threshold of QC > 20 over 5-bp window size using the Btrim program ([Kong, 2011](#_ENREF_9)). Chimeras were removed using UCHIME ([Edgar *et al.*, 2011](#_ENREF_2)), and then chimera-free sequences were clustered to generate operational taxonomic units (OTUs) with a cutoff of 97% sequence identity using UPARSE ([Edgar, 2013](#_ENREF_3)). The statistics of raw reads and reads passing the pipeline were shown in Table S2. Taxonomic classification was performed with the RDP Classifier by a confidence threshold of 0.5 ([Wang *et al.*, 2007](#_ENREF_16)). We finally rarefied the OTU table with an even-sequencing depth of 10 000.

***Statistical analyses***

The statistical methods including stepwise multiple regression analysis, gradient forest, variation partitioning (VPA), random forest analysis and structural equation models (SEM) were described as below.

First, stepwise multiple regression analysis was performed to examine the significance and predictive power of contemporary and geological variables for biological communities and ecosystem functions. The net effects of geological processes were evaluated by the improvements in the explained variances relative to those without geological processes (i.e., in the models using contemporary variables only). We used the same response variables as for piecewise linear regression analysis. The analysis was conducted with backward selection of explanatory variables for modeling ([Zhang, 2016](#_ENREF_20)). We chose the final model that met the goodness-of-fit statistics with the lowest Akaike information criterion (AIC) value ([Sakamoto *et al.*, 1986](#_ENREF_14)). ANOVA was used to test the significance of two models including or excluding geological processes as predictors, and the percent increase in the model *R*^2^ was determined as net effects of geological processes.

Second, gradient forest analysis is an extension of the random forest approach, and is an ensemble method based on regression trees that combines many decision trees to produce a distribution of splits. The standardized density of splits, that is the kernel density of splits divided by the observation density, shows where important changes in the abundance of multiple species occur along the elevational gradient and indicate the compositional change rate. We generated 2000 trees for each species of plant and bacterial communities or individual ecosystem functions. In addition, gradient forest analysis could provide a measure of conditional predictor importance for biological communities and ecosystem functions. For bacteria, we also explored the relative importance of predictors across multiple taxonomic levels from phylum to genus.

Third, VPA was used to quantify the relative contributions of contemporary and geological variables for multidiversity (MD) or multifunctionality (EMF). We partitioned explanatory variables into the following main driver categories: climate, geology (that is parent rock and weathering), local and biotic variables (Table S3). We selected explanatory variables for regression analyses by forward selection ([Miller and Farr, 1971](#_ENREF_11)). VPA was performed with the R package vegan V2.4.6 ([Oksanen *et al.*, 2017](#_ENREF_13)).

Fourth, random forest analysis was further conducted to identify the relative importance of contemporary environments and geological variables in explaining MD or EMF. Random forest is highly efficient at fitting nonparametric data, can manage various types of predictor variables, do not require prior data transformation and automatically take into account interaction effects between predictors ([Cutler *et al.*, 2007](#_ENREF_1)). Random forest is a novel machine-learning algorithm that extends standard classification and regression tree methods by creating a collection of classification trees with binary divisions ([Wei *et al.*, 2010](#_ENREF_17)). The importance measure was computed for each tree and averaged over the forest (2000 trees). This analysis was conducted using the R package randomForestSRC V2.8.0 ([Ishwaran and Kogalur, 2007](#_ENREF_7), [Ishwaran and Kogalur, 2019](#_ENREF_8)).

Finally, SEM analysis was described in detail in the main text. In addition, we constructed two alternative SEM models of MD which was computed with either species richness of bacterial phyla and plants or that of bacterial phyla. In the main text, we used the MD obtained from species richness of plants and bacteria and the richness of bacterial phyla (Fig. 4a-b) because we found similar results of SEM models of other MD values that included either species richness of bacterial phyla and plants or that of bacterial phyla for calculating MD (Fig. S20). The formulae for calculating the composite variables and the detailed modeling fit indices for these additional SEM models of MD were provided in Table S7 and S8.

**References**

Cutler DR, Edwards TC, Beard KH, Cutler A, Hess KT, Gibson JC *et al.* (2007). Random forests for classification in ecology. *Ecology* **88:** 2783-2792.

Edgar RC, Haas BJ, Clemente JC, Quince C, Knight R. (2011). UCHIME improves sensitivity and speed of chimera detection. *Bioinformatics* **27:** 2194-2200.

Edgar RC. (2013). UPARSE: highly accurate OTU sequences from microbial amplicon reads. *Nat Methods* **10:** 996-998.

Frostegård A, Bååth E. (1996). The use of phospholipid fatty acid analysis to estimate bacterial and fungal biomass in soil. *Biol Fert Soils* **22:** 59-65.

Guan Y. (1986). Soil enzyme and methodology. *Beijing: Agricultural press*.

Hill GT, Mitkowski NA, Aldrich-Wolfe L, Emele LR, Jurkonie DD, Ficke A *et al.* (2000). Methods for assessing the composition and diversity of soil microbial communities. *Appl Soil Ecol* **15:** 25-36.

Ishwaran H, Kogalur UB. (2007). Random survival forests for R. *R News* **7:** 25-31.

Ishwaran H, Kogalur UB. (2019). Fast Unified Random Forests for Survival, Regression, and Classification (RF-SRC).

Kong Y. (2011). Btrim: a fast, lightweight adapter and quality trimming program for next-generation sequencing technologies. *Genomics* **98:** 152-153.

Magoč T, Salzberg SL. (2011). FLASH: fast length adjustment of short reads to improve genome assemblies. *Bioinformatics (Oxford, England)* **27:** 2957-2963.

Miller JK, Farr SD. (1971). Bimultivariate redundancy: a comprehensive measure of interbattery relationship. *Multivar Behav Res* **6:** 313–324.

Nesbitt HW, Young GM. (1982). Early Proterozoic climates and plate motions inferred from major element chemistry of lutites. *Nature* **299:** 715-717.

Oksanen J, Blanchet FG, Kindt R, Legendre P, Minchin P, O’Hara RB *et al.* (2017). vegan: Community Ecology Package. CRAN R package.

Sakamoto Y, Ishiguro M, Kitagawa G (1986). *Akaike Information Criterion Statistics*. D. Reidel Publishing Company: Boston.

Si G, Yuan Y, Wang J, Xia Y, Lei T, Zhang G. (2014). Microbial community and soil enzyme activities along an altitudinal gradient in Sejila mountains. *Microbiology China* **41:** 2001-2011.

Wang Q, Garrity GM, Tiedje JM, Cole JR. (2007). Naive Bayesian classifier for rapid assignment of rRNA sequences into the new bacterial taxonomy. *Appl Environ Microb* **73:** 5261-5267.

Wei CL, Rowe GT, Escobar-Briones E, Boetius A, Soltwedel T, Caley MJ *et al.* (2010). Global patterns and predictions of seafloor biomass using random forests. *Plos One* **5:** e15323.

Yang H, Pancost RD, Tang C, Ding W, Dang X, Xie S. (2014). Distributions of isoprenoid and branched glycerol dialkanol diethers in Chinese surface soils and a loess–paleosol sequence: Implications for the degradation of tetraether lipids. *Org Geochem* **66:** 70-79.

Yang Y, Fang X, Galy A, Jin Z, Wu F, Yang R *et al.* (2016). Plateau uplift forcing climate change around 8.6 Ma on the northeastern Tibetan Plateau: Evidence from an integrated sedimentary Sr record. *Palaeogeography, Palaeoclimatology, Palaeoecology* **461:** 418-431.

Zhang Z. (2016). Variable selection with stepwise and best subset approaches. *Annals of Translational Medicine* **4:** 136.

**Supplementary Tables**

**Table S1**. List of 38 ecosystem functions measured. PLFA: phospholipid fatty acids. GDGT: glycerol dialkyl glycerol tetraether, including the components of isoprenoid GDGTs (iGDGT), branched GDGTs (bGDGT), GDGT-0 and Crenarchaeol.

| Group | Subgroup | Variable | Description | Unit |
| --- | --- | --- | --- | --- |
| Soil nutrients |  | nut.toc | concentration of total organic carbon | % |
|  |  | nut.tn | concentration of total nitrogen | % |
|  |  | nut.tp | concentration of total phosphate | % |
|  |  | nut.nh4 | concentration of ammonium | mg N/L |
|  |  | nut.no3 | concentration of nitrate | mg N/L |
|  |  | nut.doc | concentration of dissolved organic carbon | μg/g dry weight soil |
|  |  | nut.don | concentration of dissolved organic nitrogen | μg/g dry weight soil |
| Plant biomass | Biomass | bm.fir | biomass of fir |  |
|  | Biomass | bm.hardwood | biomass of hardwood |  |
|  | Biomass | bm.softwood | biomass of softwood |  |
|  | Biomass | bm.shrub | biomass of shrub |  |
|  | Biomass | bm.herb | biomass of herb |  |
|  | Coverage | cov.tree | coverage of tree | % |
|  | Coverage | cov.shrub | coverage of shrub | % |
|  | Coverage | cov.herb | coverage of herb | % |
|  | Height | height.tree | mean height of tree | m |
|  | Height | height.shrub | mean height of shrub | m |
|  | Height | height.herb | mean height of herb | m |
|  | Individual number | N.tree | number of tree individuals |  |
|  | Individual number | N.shrub | number of shrub individuals |  |
|  | Individual number | N.herb | number of herb individuals |  |
| Microbial biomass | PLFA | bm.plfa.bac | biomass of bacteria (bacterial PLFAs) | nmol/g |
|  | PLFA | bm.plfa.fungi | biomass of fungi (fungal PLFAs) | nmol/g |
|  | PLFA | bm.plfa.actino | biomass of Actinomycete (Actinomycete PLFAs) | nmol/g |
|  | PLFA | bm.plfa.proto | biomass of Protozoa (Protozoa PLFAs) | nmol/g |
|  | GDGT | bm.igdgt.arc | biomass of iGDGTs (from archaea) | nmol/g |
|  | GDGT | bm.bgdgt.bac | biomass of bGDGTs (from some bacteria) | nmol/g |
|  | GDGT | bm.gdgt.0 | biomass of GDGT-0 | nmol/g |
|  | GDGT | bm.cren | biomass of Crenarchaeol | nmol/g |
| Carbon cycling and storage | Enzymes activity | enzy.gluco | Glucosidase activity | μg/(g·h) |
|  | Enzymes activity | enzy.amyla | Amylase activity | μg/(g·h) |
|  | Enzymes activity | enzy.pheno | Phenoloxidase activity | μg/(g·h) |
|  | Enzymes activity | enzy.cellu | Cellulase activity | μg/(g·h) |
|  | Enzymes activity | enzy.inver | Invertase activity | μg/(g·h) |
|  | Phototrophic bacteria | relabun.rhospi | relative abundance of Rhodospirillales | % |
|  | Phototrophic bacteria | relabun.chlo | relative abundance of Chlorobi | % |
|  | Phototrophic bacteria | relabun.rhocyc | relative abundance of Rhodocyclales | % |
|  | Phototrophic bacteria | relabun.cyano | relative abundance of Cyanobacteria | % |

**Table S2**. A summary of the statistics of sequencing effort and quality control (QC) of the 16S rRNA gene sequencing data.

| Sample name | No. of reads | No. of reads passing QC | Sample name | No. of reads | No. of reads passing QC |
| --- | --- | --- | --- | --- | --- |
| 704_1^*^ | 21136 | 14009 | 2438_1 | 43515 | 30582 |
| 704_2 | 57701 | 41045 | 2438_2 | 20455 | 14145 |
| 704_3 | 66175 | 42997 | 2438_4 | 21053 | 14722 |
| 704_4 | 20565 | 14011 | 2438_5 | 48823 | 37452 |
| 704_5 | 28897 | 19432 | 2438_6 | 61680 | 40147 |
| 704_6 | 55156 | 38558 | 2438_7 | 54683 | 36462 |
| 704_7 | 25281 | 16691 | 2438_8 | 22129 | 14540 |
| 704_8 | 46218 | 32965 | 2438_9 | 29454 | 20415 |
| 704_9 | 24455 | 15948 | 2438_10 | 45609 | 32623 |
| 704_10 | 26967 | 17873 | 2638_1 | 39896 | 26851 |
| 877_1 | 29428 | 20210 | 2638_2 | 32079 | 21455 |
| 877_2 | 20373 | 14078 | 2638_3 | 34890 | 23030 |
| 877_3 | 28101 | 17240 | 2638_4 | 25743 | 16401 |
| 877_4 | 31841 | 19866 | 2638_5 | 23009 | 17708 |
| 877_5 | 50132 | 37323 | 2638_6 | 45301 | 29856 |
| 877_6 | 63738 | 41879 | 2638_7 | 41394 | 26745 |
| 877_7 | 64409 | 41611 | 2638_8 | 54758 | 38054 |
| 877_8 | 20758 | 14126 | 2638_9 | 23548 | 16113 |
| 877_9 | 55745 | 39448 | 2638_10 | 29606 | 19824 |
| 877_10 | 23896 | 15324 | 2743_1 | 30131 | 20983 |
| 1096_1 | 73843 | 47413 | 2743_2 | 24016 | 16363 |
| 1096_2 | 63805 | 40470 | 2743_3 | 47885 | 33541 |
| 1096_3 | 25502 | 18428 | 2743_4 | 27660 | 18570 |
| 1096_4 | 20775 | 14780 | 2743_5 | 43335 | 29314 |
| 1096_5 | 58964 | 37971 | 2743_6 | 35655 | 22995 |
| 1096_6 | 27278 | 19220 | 2743_7 | 41972 | 27496 |
| 1096_7 | 50990 | 31971 | 2743_8 | 35059 | 23386 |
| 1096_8 | 34473 | 22606 | 2743_9 | 34548 | 22812 |
| 1096_9 | 52296 | 32973 | 2743_10 | 26143 | 16485 |
| 1096_10 | 54947 | 36160 | 2964_1 | 41245 | 27589 |
| 1397_1 | 22069 | 13670 | 2964_2 | 44242 | 31038 |
| 1397_2 | 23061 | 14838 | 2964_3 | 39138 | 27385 |
| 1397_3 | 62886 | 41079 | 2964_4 | 58361 | 38357 |
| 1397_4 | 19653 | 12855 | 2964_5 | 39176 | 26051 |
| 1397_5 | 19857 | 13097 | 2964_6 | 39666 | 27686 |
| 1397_6 | 22270 | 14639 | 2964_7 | 42432 | 26328 |
| 1397_7 | 33217 | 20786 | 2964_8 | 51385 | 35732 |
| 1397_8 | 52701 | 37322 | 2964_9 | 55709 | 38716 |
| 1397_9 | 22212 | 13917 | 2964_10 | 56377 | 36316 |
| 1397_10 | 74475 | 47040 | 3144_1 | 86216 | 54427 |
| 1546_1 | 48043 | 32368 | 3144_2 | 42266 | 29243 |
| 1546_2 | 57907 | 37468 | 3144_3 | 24901 | 16305 |
| 1546_3 | 25848 | 18034 | 3144_4 | 45709 | 30759 |
| 1546_4 | 55415 | 41821 | 3144_5 | 37700 | 24279 |
| 1546_5 | 39380 | 28948 | 3144_6 | 45957 | 30753 |
| 1546_6 | 52999 | 39707 | 3144_7 | 32791 | 20092 |
| 1546_7 | 50046 | 36893 | 3144_8 | 33152 | 21540 |
| 1546_8 | 22250 | 15600 | 3144_9 | 33400 | 22110 |
| 1546_9 | 52078 | 34219 | 3144_10 | 25182 | 17008 |
| 1546_10 | 39967 | 29233 | 3244_1 | 25376 | 17022 |
| 1669_1 | 70031 | 45960 | 3244_2 | 31981 | 20392 |
| 1669_2 | 61123 | 33545 | 3244_3 | 33274 | 22008 |
| 1669_3 | 71928 | 47104 | 3244_4 | 33655 | 21001 |
| 1669_4 | 64383 | 37483 | 3244_5 | 32377 | 20586 |
| 1669_5 | 75778 | 51131 | 3244_6 | 37846 | 23962 |
| 1669_6 | 65684 | 39908 | 3244_7 | 43523 | 29253 |
| 1669_7 | 67400 | 41534 | 3244_8 | 35930 | 23757 |
| 1669_8 | 61288 | 37808 | 3244_9 | 41995 | 27920 |
| 1669_9 | 66851 | 42104 | 3244_10 | 51941 | 34756 |
| 1669_10 | 25348 | 10946 | 3419_1 | 38749 | 25369 |
| 1893_1 | 53575 | 38485 | 3419_2 | 35517 | 24286 |
| 1893_2 | 58103 | 41077 | 3419_3 | 31428 | 21132 |
| 1893_3 | 78216 | 49855 | 3419_4 | 27489 | 18433 |
| 1893_4 | 55711 | 33794 | 3419_5 | 28614 | 18813 |
| 1893_5 | 22235 | 14194 | 3419_6 | 30776 | 20754 |
| 1893_6 | 21444 | 13712 | 3419_7 | 31050 | 22226 |
| 1893_7 | 33004 | 21437 | 3419_8 | 30917 | 21226 |
| 1893_8 | 64890 | 42560 | 3419_9 | 37810 | 25440 |
| 1893_9 | 70004 | 43957 | 3419_10 | 27730 | 19419 |
| 1893_10 | 53868 | 34617 | 3629_1 | 45907 | 30947 |
| 2166_1 | 63288 | 43783 | 3629_2 | 24476 | 16560 |
| 2166_2 | 56180 | 38750 | 3629_3 | 33545 | 22050 |
| 2166_3 | 49722 | 37583 | 3629_4 | 40197 | 27607 |
| 2166_4 | 60713 | 39922 | 3629_5 | 39189 | 26922 |
| 2166_5 | 58780 | 40209 | 3629_6 | 57466 | 40743 |
| 2166_6 | 77110 | 51500 | 3629_7 | 38037 | 26600 |
| 2166_7 | 70970 | 46590 | 3629_8 | 40893 | 27527 |
| 2166_8 | 64431 | 43166 | 3629_9 | 26478 | 18494 |
| 2166_9 | 53018 | 31677 | 3629_10 | 44125 | 30774 |
| 2166_10 | 63215 | 39230 | 3760_1 | 35659 | 25675 |
| 2293_1 | 53091 | 36414 | 3760_2 | 42646 | 30104 |
| 2293_2 | 71138 | 44819 | 3760_3 | 40847 | 28580 |
| 2293_3 | 21428 | 14687 | 3760_4 | 29565 | 22256 |
| 2293_4 | 58523 | 38448 | 3760_5 | 39789 | 27695 |
| 2293_5 | 59515 | 39610 | 3760_6 | 40129 | 28214 |
| 2293_6 | 56317 | 32559 | 3760_7 | 46506 | 32357 |
| 2293_7 | 50420 | 35221 | 3760_8 | 42710 | 31060 |
| 2293_8 | 52659 | 34955 | 3760_9 | 25374 | 17521 |
| 2293_9 | 50289 | 33322 | 3760_10 | 55802 | 36538 |
| 2293_10 | 19775 | 13573 |  |  |  |

^*^ For each of sample name, we used the abbreviation by the combination of “elevation” and “replication”. For instance, the abbreviation “704_1” stands for the sample from the first replication at the elevation of 704 m.

**Table S3**. Explanatory variables used to explain biological communities and ecosystem functions. These variables were considered based on our conceptual framework in Fig. 1a: climate, local and biotic variables as contemporary environments, and parent rock and weathering variables as geological processes. PCA: principal component analysis; DCA: detrended correspondence analysis; CIA: chemical index of alteration.

|  | Group | Variable | Description |
| --- | --- | --- | --- |
| Geological processes | Parent rock | Quartz | mass concentration of quartz |
|  |  | Plagioclase | mass concentration of plagioclase |
|  |  | K-feldspar | mass concentration of K-feldspar |
|  |  | Amphibole | mass concentration of amphibole |
|  |  | Muscovite | mass concentration of muscovite |
|  |  | Chlorite | mass concentration of chlorite |
|  |  | Mineral.pc1 | PCA axis 1 of minerals |
|  |  | Mineral.pc2 | PCA axis 2 of minerals |
|  | Weathering | CIA | CIA index |
|  |  | Mg/Al | Mg/Al ratio |
|  |  | Ca/Al | Ca/Al ratio |
|  |  | Ti/Fe | Ti/Fe ratio |
|  |  | Ti/Al | Ti/Al ratio |
|  |  | Metal.pc1 | PCA axis 1 of metal elements |
|  |  | Metal.pc2 | PCA axis 2 of metal elements |
| Contemporary processes | Climate | Temp | mean annual temperature |
|  |  | Precip | mean annual precipitation |
|  | Local | pH | soil pH |
|  |  | Moisture | soil moisture |
|  | Biotic | Veg.rich | species richness of plant |
|  |  | Bac.rich | species richness of bacteria |
|  |  | phylum.rich^*^ | species richness of each of 18 bacterial phyla |
|  |  | Veg.dca1 | DCA axis 1 of plant |
|  |  | Veg.dca2 | DCA axis 2 of plant |
|  |  | Bac.dca1 | DCA axis 1 of bacteria |
|  |  | Bac.dca2 | DCA axis 2 of bacteria |

^*^ When specific phylum was considered, we used the abbreviation by the combination of “phylum name” and “.rich”. For instance, the abbreviation “proteobacteria.rich” stands for the species richness of the phylum proteobacteria.

**Table S4**. Formulae to calculate composite variables for structure equation models (SEMs) of multidiversity (MD) and ecosystem multifunctionality (EMF). The obtained composite variables were used in Fig. 4. The abbreviations of included variables are listed in Table S3.

| Response | Composite | Formula |
| --- | --- | --- |
| MD | Climate | Climate=8.722e-01*Temp-5.325e-01*Precip |
| MD | Rock | Rock=1.238e-01*Plagioclase+6.509e-01*Muscovite+1.833e-01*Chlorite |
| MD | Weathering | Weathering=5.352e-01*Ca/Al-4.631e-01*Ti/Fe+3.264e-01*Metal.pc1-1.898e-01*Metal.pc2 |
| MD | Local | Local=7.789e-01*pH |
| EMF | Climate | Climate=-8.791e-01*tTemp+8.297e-01*Precip |
| EMF | Rock | Rock=3.892e-01*Quartz+2.874e-01*Plagioclase+1.128e-01*Amphibole |
| EMF | Weathering | Weathering=7.267e-01*CIA-1.619e+00*Mg/Al+1.883e+00*Ca/Al+1.758e-01*Ti/Fe |
| EMF | Local | Local=7.263e-01*Moisture |
| EMF | Biotic | Biotic=-1.689e-01*veg.dca2+1.641e-01*bac.dca2+2.191e-01*Acidobacteria.rich-3.127e-01*Firmicutes.rich+3.310e-01*Gammaproteobacteria.rich+6.302e-01*Actinobacteria.rich-3.792e-01*Bacteroidetes.rich-2.998e-01*Chloroflexi.rich-1.827e-01*Cyanobacteria.rich-3.400e-01*Gemmatimonadetes.rich-3.898e-01*WS3.rich+1.521e-01*Spirochaetes.rich-1.836e-01*BRC1.rich |

**Table S5**. Summary of the model fit statistics evaluated for the standardized structural equation model (SEM). We examined the effects of predictor variables on multidiversity (MD) or ecosystem multifunctionality (EMF) by excluding or including geological variables, and the best-fitting models were shown in Fig. 4. We constructed the full SEM models based on our conceptual framework, and further performed sequential models by dropping non-significant paths from the full models. χ^2^: Chi-square. *P*: p-value of chi-square test. df: degrees of freedom. CFI: comparative fit index. SRMR: standardized root mean squared residual. AIC_c_: second-order Akaike information criterion. ΔAIC_c_: delta AIC_c_.

| SEM model | Response | Omitted paths | df | χ^2^ | *P* | CFI | SRMR | AIC_c_ | ΔAIC_c_ |
| --- | --- | --- | --- | --- | --- | --- | --- | --- | --- |
| MD without geological variables | | |  |  |  |  |  |  |  |
| 1^a^ | MD |  | 0 | 0 | 0 | 1 | 0 | 873.5 | 1.48 |
| 2^b^ | MD | Climate -> MD | 1 | 0.587 | 0.443 | 1 | 0.012 | 872.0 | 0 |
| MD with geological variables | | |  |  |  |  |  |  |  |
| 1^a^ | MD |  | 0 | 0 | 0 | 1 | 0 | 1352.4 | 2.05 |
| 2^b^ | MD | Climate -> MD | 1 | 0.194 | 0.659 | 1 | 0.004 | 1350.3 | 0 |
| 3 | MD | Climate -> MD;  Climate -> Weathering | 2 | 2.763 | 0.251 | 0.998 | 0.037 | 1350.7 | 0.35 |
| EMF without geological variables | | |  |  |  |  |  |  |  |
| 1^a^ | EMF |  | 0 | 0 | 0 | 1 | 0 | 827.9 | 4.15 |
| 2 | EMF | Climate -> EMF | 1 | 0.033 | 0.856 | 1 | 0.003 | 825.7 | 2.01 |
| 3^b^ | EMF | Climate -> EMF;  Climate -> Biotic | 2 | 0.166 | 0.92 | 1 | 0.008 | 823.7 | 0 |
| EMF with geological variables | | |  |  |  |  |  |  |  |
| 1^a^ | EMF |  | 0 | 0 | 0 | 1 | 0 | 1132.9 | 9.14 |
| 2 | EMF | Rock -> Biotic | 1 | 0.061 | 0.804 | 1 | 0.003 | 1130.5 | 6.72 |
| 3 | EMF | Rock -> Biotic;  Climate -> Biotic | 2 | 0.785 | 0.675 | 1 | 0.012 | 1128.8 | 5.01 |
| 4 | EMF | Rock -> Biotic;  Climate -> Biotic;  Rock -> Local | 3 | 1.654 | 0.647 | 1 | 0.017 | 1127.3 | 3.48 |
| 5 | EMF | Rock -> Biotic;  Climate -> Biotic;  Rock -> Local;  Climate -> Rock | 3 | 1.654 | 0.647 | 1 | 0.017 | 1126.9 | 3.11 |
| 6 | EMF | Rock -> Biotic;  Climate -> Biotic;  Rock -> Local;  Climate -> Rock;  Climate -> EMF | 4 | 2.039 | 0.729 | 1 | 0.019 | 1124.9 | 1.16 |
| 7^b^ | EMF | Rock -> Biotic;  Climate -> Biotic;  Rock -> Local;  Climate -> Rock;  Climate -> EMF;  Rock -> EMF | 5 | 3.177 | 0.673 | 1 | 0.024 | 1123.8 | 0 |

^a^ Full SEM models; ^b^ Best-fitting models shown in red.

**Table S6**. The breakpoint estimation within the 1800–3000 m elevation ranges for plants, bacteria, ecosystem functions and drivers using piecewise regression analyses. DCA: detrended correspondence analysis; PCA: principal component analysis; EMF: ecosystem multifunctionality; PLFA: phospholipid fatty acids. GDGT: glycerol dialkyl glycerol tetraether.

| Groups | Facets | Variables | Description | Breakpoint (m) |
| --- | --- | --- | --- | --- |
| Plants | Diversity | herb.rich | richness of herb | 1893 |
| Plants | Diversity | tree.rich | richness of tree | 2022 |
| Plants | Compsition (Comp) | herb.dca1 | DCA axis 1 of herb | 2260 |
| Plants | Compsition (Comp) | tree.dca1 | DCA axis 1 of tree | 2319 |
| Plants | Compsition (Comp) | tree.dca2 | DCA axis 2 of tree | 1883 |
| Plants | Relative abundance (RA) | herb.pct | percentage of herb | 1893 |
| Plants | Relative abundance (RA) | shrub.pct | percentage of shrub | 2001 |
| Plants | Relative abundance (RA) | tree.pct | percentage of tree | 2743 |
| Plants | Relative abundance (RA) | Smilax | RA of Smilax | 2214 |
| Plants | Relative abundance (RA) | Gaultheria | RA of Gaultheria | 2420 |
| Plants | Relative abundance (RA) | Ribes | RA of Ribes | 2275 |
| Plants | Relative abundance (RA) | Camellia | RA of Camellia | 1893 |
| Plants | Relative abundance (RA) | Synotis | RA of Synotis | 2568 |
| Plants | Relative abundance (RA) | Juncus | RA of Juncus | 2965 |
| Plants | Relative abundance (RA) | Maesa | RA of Maesa | 2602 |
| Plants | Relative abundance (RA) | Rhododendron | RA of Rhododendron | 1858 |
| Plants | Relative abundance (RA) | Schefflera | RA of Schefflera | 2507 |
| Plants | Relative abundance (RA) | Coniogramme | RA of Coniogramme | 2743 |
| Plants | Relative abundance (RA) | Gramineae | RA of Gramineae | 2743 |
| Plants | Relative abundance (RA) | Piper | RA of Piper | 1803 |
| Plants | Relative abundance (RA) | Daphniphyllum | RA of Daphniphyllum | 2557 |
| Plants | Relative abundance (RA) | Sorbus | RA of Sorbus | 2161 |
| Plants | Relative abundance (RA) | Polygonatum | RA of Polygonatum | 2293 |
| Plants | Relative abundance (RA) | Castanopsis | RA of Castanopsis | 2862 |
| Plants | Relative abundance (RA) | Pilea | RA of Pilea | 2166 |
| Plants | Relative abundance (RA) | Eurya | RA of Eurya | 2743 |
| Plants | Relative abundance (RA) | Circaea | RA of Circaea | 2268 |
| Plants | Relative abundance (RA) | Aeschynanthus | RA of Aeschynanthus | 2539 |
| Plants | Relative abundance (RA) | Equisetum | RA of Equisetum | 2615 |
| Plants | Relative abundance (RA) | Lyonia | RA of Lyonia | 2293 |
| Plants | Relative abundance (RA) | Meliosma | RA of Meliosma | 1956 |
| Plants | Relative abundance (RA) | Rubiaceae | RA of Rubiaceae | 2228 |
| Plants | Relative abundance (RA) | Cyclobalanopsis | RA of Cyclobalanopsis | 1819 |
| Plants | Relative abundance (RA) | Lonicera | RA of Lonicera | 2291 |
| Plants | Relative abundance (RA) | Bulbophyllum | RA of Bulbophyllum | 2638 |
| Plants | Relative abundance (RA) | Saurauia | RA of Saurauia | 2661 |
| Plants | Relative abundance (RA) | Tsuga | RA of Tsuga | 2638 |
| Plants | Relative abundance (RA) | Myrsine | RA of Myrsine | 2293 |
| Plants | Relative abundance (RA) | Calanthe | RA of Calanthe | 2733 |
| Plants | Relative abundance (RA) | Neillia | RA of Neillia | 2743 |
| Plants | Relative abundance (RA) | Macaranga | RA of Macaranga | 2108 |
| Plants | Relative abundance (RA) | Rhaphidophora | RA of Rhaphidophora | 2282 |
| Plants | Relative abundance (RA) | Tetrastigma | RA of Tetrastigma | 2638 |
| Plants | Relative abundance (RA) | Vaccinium | RA of Vaccinium | 2881 |
| Plants | Relative abundance (RA) | Rutaceae | RA of Rutaceae | 2767 |
| Plants | Relative abundance (RA) | Bambusoideae | RA of Bambusoideae | 2716 |
| Plants | Relative abundance (RA) | Boehmeria | RA of Boehmeria | 2166 |
| Bacteria | Diversity | Acidobacteria.rich | richness of Acidobacteria | 2964 |
| Bacteria | Diversity | Armatimonadetes.rich | richness of Armatimonadetes | 2638 |
| Bacteria | Diversity | Cyanobacteria.rich | richness of Cyanobacteria | 2293 |
| Bacteria | Diversity | Deltaproteobacteria.rich | richness of Deltaproteobacteria | 2964 |
| Bacteria | Diversity | Planctomycetes.rich | richness of Acidobacteria | 2438 |
| Bacteria | Diversity | Verrucomicrobia.rich | richness of Acidobacteria | 2638 |
| Bacteria | Compsition (Comp) | bac.dca2 | DCA axis 2 of total bacteria | 2964 |
| Bacteria | Compsition (Comp) | BRC1.dca1 | DCA axis 1 of BRC1 | 1968 |
| Bacteria | Compsition (Comp) | Cyanobacteria.dca1 | DCA axis 1 of Cyanobacteria | 1836 |
| Bacteria | Compsition (Comp) | Spirochaetes.dca1 | DCA axis 1 of Spirochaetes | 1983 |
| Bacteria | Compsition (Comp) | gdgt.dca1 | DCA axis 1 of GDGTs | 2748 |
| Bacteria | Compsition (Comp) | plfa.dca2 | DCA axis 2 of PLFAs | 2638 |
| Bacteria | Relative abundance (RA) | relabun.Alpha | RA of Alphaproteobacteria | 2879 |
| Bacteria | Relative abundance (RA) | relabun.Delta | RA of Deltaproteobacteria | 2743 |
| Functions | EMF | EMF | ecosystem multifunctionality | 2293 |
| Functions | EMF | EMF.bm | EMF of plant and microbial biomass | 2293 |
| Functions | EMF | EMF.bm.veg | EMF of plant biomass | 2352 |
| Functions | EMF | EMF.nut | EMF of soil nutrient | 2638 |
| Functions | Individual (Indiv) | bm.hardwood | biomass of hardwood | 2166 |
| Functions | Individual (Indiv) | bm.shrub | biomass of shrub | 1876 |
| Functions | Individual (Indiv) | bm.herb | biomass of herb | 1830 |
| Functions | Individual (Indiv) | bm.plfa.actino | PLFA of Actinomycete | 2438 |
| Functions | Individual (Indiv) | bm.plfa.bac | PLFA of bacteria | 2293 |
| Functions | Individual (Indiv) | bm.plfa.fungi | PLFA of fungi | 2638 |
| Functions | Individual (Indiv) | cov.shrub | coverage of shrub | 2015 |
| Functions | Individual (Indiv) | cov.herb | coverage of herb | 1893 |
| Functions | Individual (Indiv) | enzy.Amyla | Amylase activity | 2166 |
| Functions | Individual (Indiv) | enzy.gluco | Glucosidase activity | 2293 |
| Functions | Individual (Indiv) | height.herb | mean height of herb | 2330 |
| Functions | Individual (Indiv) | height.tree | mean height of tree | 2259 |
| Functions | Individual (Indiv) | N.shrub | number of shrub individuals | 2406 |
| Functions | Individual (Indiv) | nut.toc | concentration of total organic carbon | 2964 |
| Functions | Individual (Indiv) | nut.tp | concentration of total phosphate | 2906 |
| Functions | Individual (Indiv) | nut.doc | concentration of dissolved organic carbon | 2964 |
| Functions | Individual (Indiv) | nut.nh4 | concentration of ammonium | 2261 |
| Functions | Individual (Indiv) | nut.no3 | concentration of nitrate | 2539 |
| Functions | Individual (Indiv) | relabun.chlo | RA of Chlorobi | 2347 |
| Functions | Individual (Indiv) | relabun.cyano | RA of Cyanobacteria | 2638 |
| Functions | Individual (Indiv) | relabun.rhocyc | RA of Rhodocyclales | 2743 |
| Functions | Individual (Indiv) | relabun.rhospi | RA of Rhodospirillales | 2964 |
| Functions | Compsition (Comp) | funs.dca1 | DCA axis 1 of functions | 1893 |
| Functions | Compsition (Comp) | fun.bm.bac.dca1 | DCA axis 1 of bacterial biomass | 1893 |
| Functions | Compsition (Comp) | fun.bm.dca1 | DCA axis 1 of biomass | 2166 |
| Functions | Compsition (Comp) | fun.bm.dca2 | DCA axis 2 of biomass | 2293 |
| Functions | Compsition (Comp) | fun.bm.veg.dca1 | DCA axis 1 of plant biomass | 2132 |
| Functions | Compsition (Comp) | fun.bm.veg.dca2 | DCA axis 2 of plant biomass | 1921 |
| Functions | Compsition (Comp) | fun.enzy.pb.dca2 | DCA axis 2 of carbon cycling and storage | 2293 |
| Functions | Compsition (Comp) | fun.nut.pc2 | PCA axis 2 of soil nutrient | 2616 |
| Functions | Compsition (Comp) | fun.pb.dca1 | DCA axis 1 of phototrophic bacteria | 2743 |
| Drivers | Climate | precip | mean annual precipitation | 1820 |
| Drivers | Parent rock (Rock) | quartz | mass concentration of quartz | 2964 |
| Drivers | Parent rock (Rock) | K.feldspar | mass concentration of K-feldspar | 2964 |
| Drivers | Parent rock (Rock) | muscovite | mass concentration of muscovite | 1838 |
| Drivers | Parent rock (Rock) | mineral.pc2 | PCA axis 2 of minerals | 1865 |
| Drivers | Weathering | ca.al.ratio | Ca/Al ratio | 1893 |
| Drivers | Weathering | mg.al.ratio | Mg/Al ratio | 2125 |
| Drivers | Weathering | ti.al.ratio | Ti/Al ratio | 2638 |
| Drivers | Weathering | metal.pc1 | PCA axis 1 of metal elements | 2964 |

**Table S7**. Formulae to calculate composite variables for alternative structure equation models of multidiversity (MD). The obtained composite variables were used in Fig. S20. The abbreviations of included variables are listed in Table S3.

| Response | Composite | Formula |
| --- | --- | --- |
| MD^a^ | Climate | Climate=8.714e-01*Temp-5.321e-01*Precip |
| MD^a^ | Rock | Rock=1.236e-01*Plagioclase+6.499e-01*Muscovite+1.841e-01*Chlorite |
| MD^a^ | Weathering | Weathering=5.282e-01*Ca/Al-4.621e-01*Ti/Fe+3.192e-01*Metal.pc1-1.916e-01*Metal.pc2 |
| MD^a^ | Local | Local=7.766e-01*pH |
| MD^b^ | Climate | Climate=7.592e-01*Temp-4.461e-01*Pprecip |
| MD^b^ | Rock | Rock=1.975e-01*Plagioclase+1.232e-01*K-feldspar+1.373e-01*Amphibole+7.410e-01*Muscovite+1.769e-01*Chlorite |
| MD^b^ | Weathering | Weathering=4.783e-01*Ca/Al-4.631e-01*Ti/Fe+2.918e-01*Metal.pc1-2.191e-01*Metal.pc2 |
| MD^b^ | Local | Local=7.391e-01*pH-9.069e-02*Moisture |

^a^ Models of MD calculated with species richness of bacterial phyla and plants;

^b^ Models of MD calculated with species richness of bacterial phyla.

**Table S8**. Summary of the model fit statistics evaluated for the alternative structural equation model (SEM). We examined the effects of predictor variables on multidiversity (MD) by excluding or including geological variables, and the best-fitting models were shown in Fig. S20. We constructed the full SEM models based on our conceptual framework, and further performed sequential models by dropping non-significant paths from the full models. χ^2^: Chi-square. *P*: p-value of chi-square test. df: degrees of freedom. CFI: comparative fit index. SRMR: standardized root mean squared residual. AIC_c_: second-order Akaike information criterion. ΔAIC_c_: delta AIC_c_.

| SEM model | Response | Omitted paths | df | χ^2^ | *P* | CFI | SRMR | AIC_c_ | ΔAIC_c_ |
| --- | --- | --- | --- | --- | --- | --- | --- | --- | --- |
| MD^c^ without geological variables | | |  |  |  |  |  |  |  |
| 1^a^ | MD^c^ |  | 0 | 0 | 0 | 1 | 0 | 873.6 | 1.51 |
| 2^b^ | MD^c^ | Climate -> MD | 1 | 0.555 | 0.456 | 1 | 0.012 | 872.1 | 0 |
| MD^c^ with geological variables | | |  |  |  |  |  |  |  |
| 1^a^ | MD^c^ |  | 0 | 0 | 0 | 1 | 0 | 1351.5 | 2.03 |
| 2^b^ | MD^c^ | Climate -> MD | 1 | 0.212 | 0.645 | 1 | 0.004 | 1349.4 | 0 |
| 3 | MD^c^ | Climate -> MD;  Climate -> Weathering | 2 | 2.799 | 0.247 | 1 | 0.037 | 1349.8 | 0.37 |
| MD^d^ without geological variables | | |  |  |  |  |  |  |  |
| 1^a^ | MD^d^ |  | 0 | 0 | 0 | 1 | 0 | 837.4 | 0 |
| 2^b^ | MD^d^ | Climate -> MD | 1 | 2.616 | 0.106 | 0.99 | 0.026 | 838.0 | 0.55 |
| MD^d^ with geological variables | | |  |  |  |  |  |  |  |
| 1^a^ | MD^d^ |  | 0 | 0 | 0 | 1 | 0 | 1313.7 | 2.11 |
| 2^b^ | MD^d^ | Climate -> MD | 1 | 0.13 | 0.719 | 1 | 0.003 | 1311.6 | 0 |
| 3 | MD^d^ | Climate -> MD;  Climate -> Weathering | 2 | 3.182 | 0.204 | 1 | 0.036 | 1312.5 | 0.83 |

^a^ Full SEM models; ^b^ Best-fitting models shown in red;

^c^ Models of MD calculated with species richness of bacterial phyla and plants;

^d^ Models of MD calculated with species richness of bacterial phyla.

**Table S9**. The direct, indirect and total effects of predictor variables on multidiversity (MD) and ecosystem multifunctionality (EMF). These effects are related to the best-fitting standardized structural equation models (SEMs) as shown in Fig. 4. Predictor variables include climate, parent rock, weathering, local and biotic attributes.

| SEM model | Predictor | Response | Direct | Indirect | Total |
| --- | --- | --- | --- | --- | --- |
| Excluding geology | Climate | MD | 0.000 | 0.414 | 0.414 |
|  | Local | MD | 0.779 | 0.000 | 0.779 |
| Including geology | Climate | MD | 0.000 | 0.315 | 0.315 |
|  | Rock | MD | 0.123 | 0.381 | 0.504 |
|  | Weathering | MD | 0.302 | 0.238 | 0.540 |
|  | Local | MD | 0.531 | 0.000 | 0.531 |
| Excluding geology | Climate | EMF | 0.000 | 0.218 | 0.218 |
|  | Local | EMF | 0.521 | 0.205 | 0.726 |
|  | Biotic | EMF | 0.415 | 0.000 | 0.415 |
| Including geology | Climate | EMF | 0.000 | 0.233 | 0.233 |
|  | Rock | EMF | 0.000 | 0.343 | 0.343 |
|  | Weathering | EMF | 0.164 | 0.426 | 0.590 |
|  | Local | EMF | 0.472 | 0.085 | 0.557 |
|  | Biotic | EMF | 0.341 | 0.000 | 0.341 |

**Supplementary Figures**


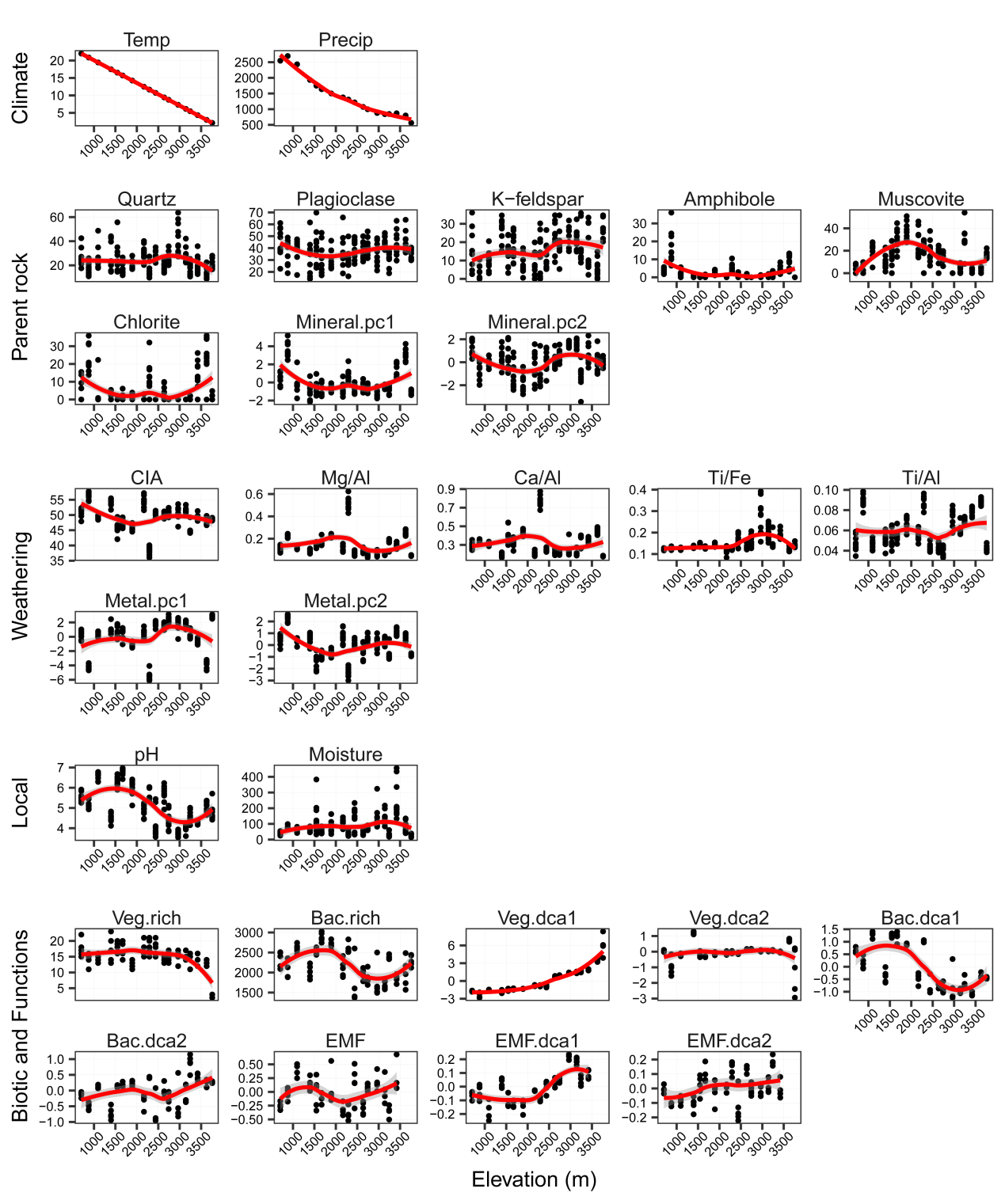


**Figure S1**. Elevational patterns in ecosystem properties visualized with loess regression models. These ecosystem properties included the explanatory variables associated with climate, parent rock, weathering, local and biotic attributes as well as ecosystem functions. Detailed information about explanatory variables is listed in Table S3. The shaded region represents 95% confidence on the regression estimates. For better visualization, we only selected several biotic and function variables, such as species richness, detrended correspondence analyses (DCA) of biological communities for plants and bacteria, ecosystem multifunctionality (EMF), and DCA of ecosystem functions.

**
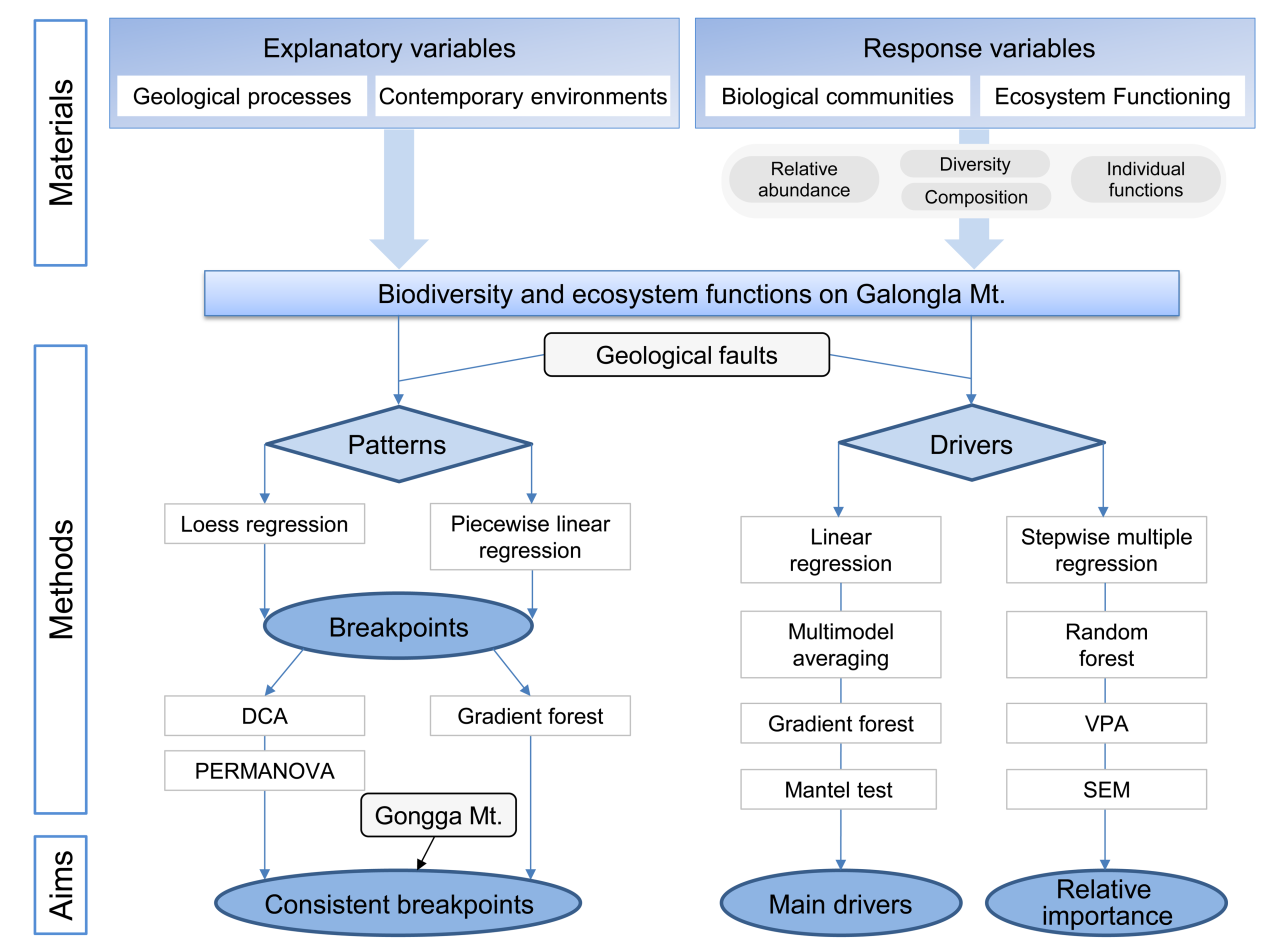
**

**Figure S2**. The framework regarding “Materials, Methods and Aims”. Mt.: Mountain. DCA: Detrended correspondence analysis. VPA: Variation partitioning analysis. SEM: Structural equation model.


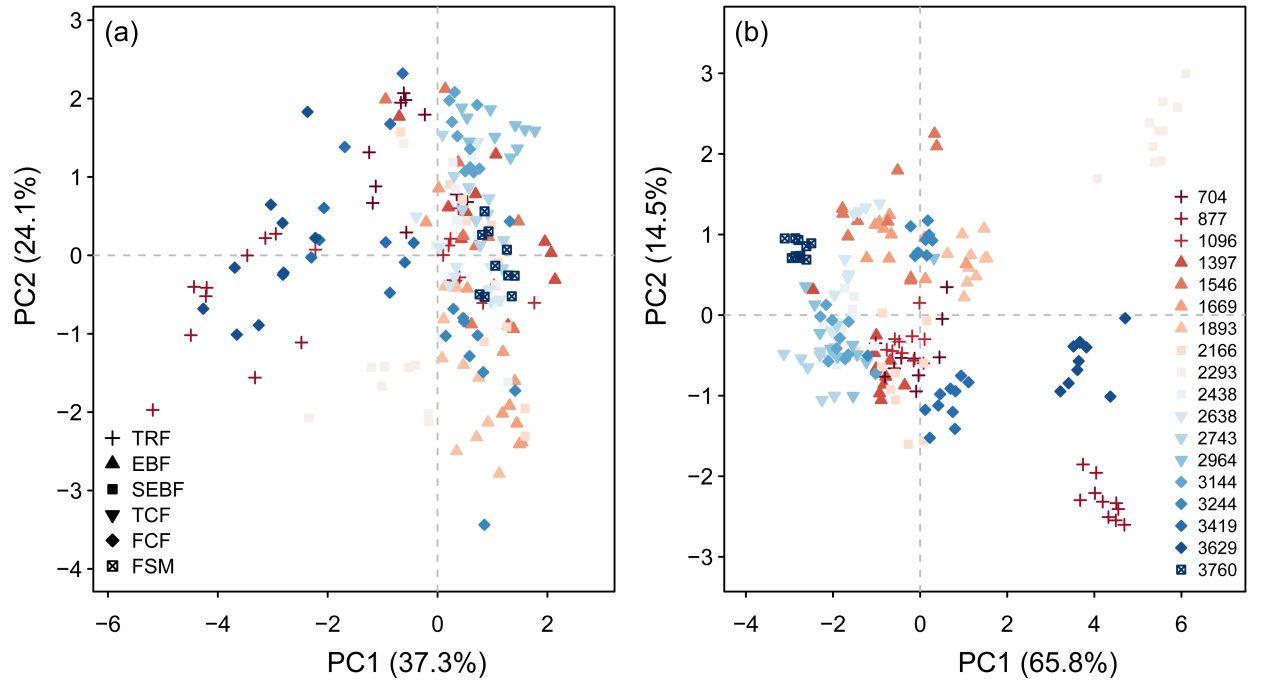


**Figure S3**. Principal component analysis (PCA) of compositions of minerals (a) and metal elements (b). Vegetation zones comprise tropical monsoon rain forest (TRF), subtropical evergreen broadleaved forest (EBF), subtropical evergreen and semi-evergreen broadleaved forest (SEBF), temperate mixed coniferous broadleaved forest (TCF), frigid-temperate coniferous forest (FCF) and frigid shrub meadows (FSM).


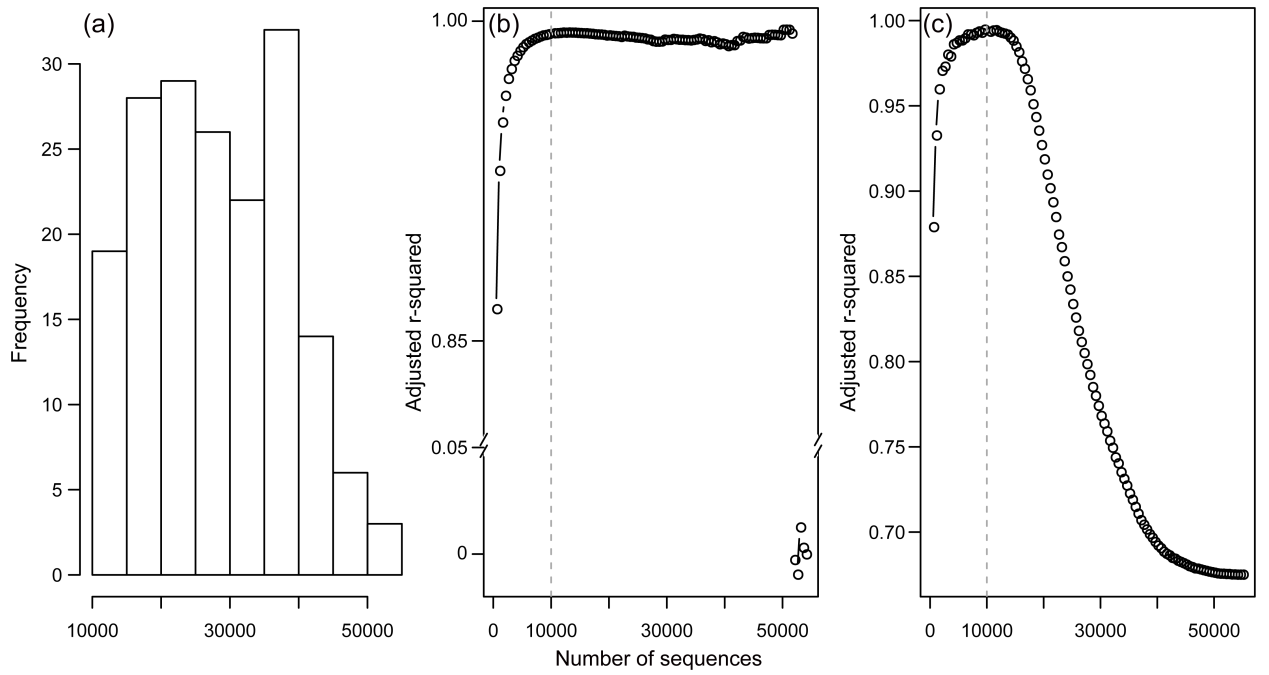


**Figure S4**. The sequencing depth and the effects of sequencing depth on bacterial diversity estimates.

(a) The frequency of sequencing depth across the 180 samples. The lowest sequencing depth was 10946 sequences.

(b, c) The relationships between species richness rarefied at 10000 sequences and those at the other sequencing depths. These sequencing depths varied from 500 to 55000 sequences per sample with a step of 500, which is generated via the rarefaction approach. We applied two scenarios to test the effects of sequencings depths on the measured species richness.

(b) One scenario is to keep even-sampling across all possible samples. Specifically, we applied the rarefaction approach for all samples across the depth from 500 to 55000 sequences. All 180 samples were kept in the diversity measurement when the even-sampling depth was below 10946, but some samples will be discarded afterwards. In this way, we could always calculate the diversity with the same sequencing depths for some of these 180 samples.

(c) The other scenario is to keep all the samples across the rarefying steps. Specifically, when the samples with lower sequence numbers than the desired rarefied sequencing depth (for instance, there were 47 samples with the sequencing depths lower than a depth of 20000 sequences), their actual sequencing depths would be applied. In this way, we produced even-sampling efforts for all samples below the depth of 10946 sequences, but the heterogeneity of sequencing among samples will increase afterwards.

For each rarefying step of the above two scenarios, we used linear models to obtain the adjusted R^2^ for the relationships between species richness rarefied at 10000 sequences and those at the other sequencing depths. The obtained values of adjusted R^2^, indicated with open circles, were plotted against their sequencing depths (b, c). We could see the sequencing depths below 6000 would strongly affect the estimated diversity, but such effects were minimized and consistent afterwards (b). This finding support that our depth at 10000 sequences could produce reliable diversity estimates for these 180 samples. As we expected, we found the strong effects of sequencing heterogeneity on the estimated diversity (c), which further supports the necessary procedure in applying rarefaction for community studies.


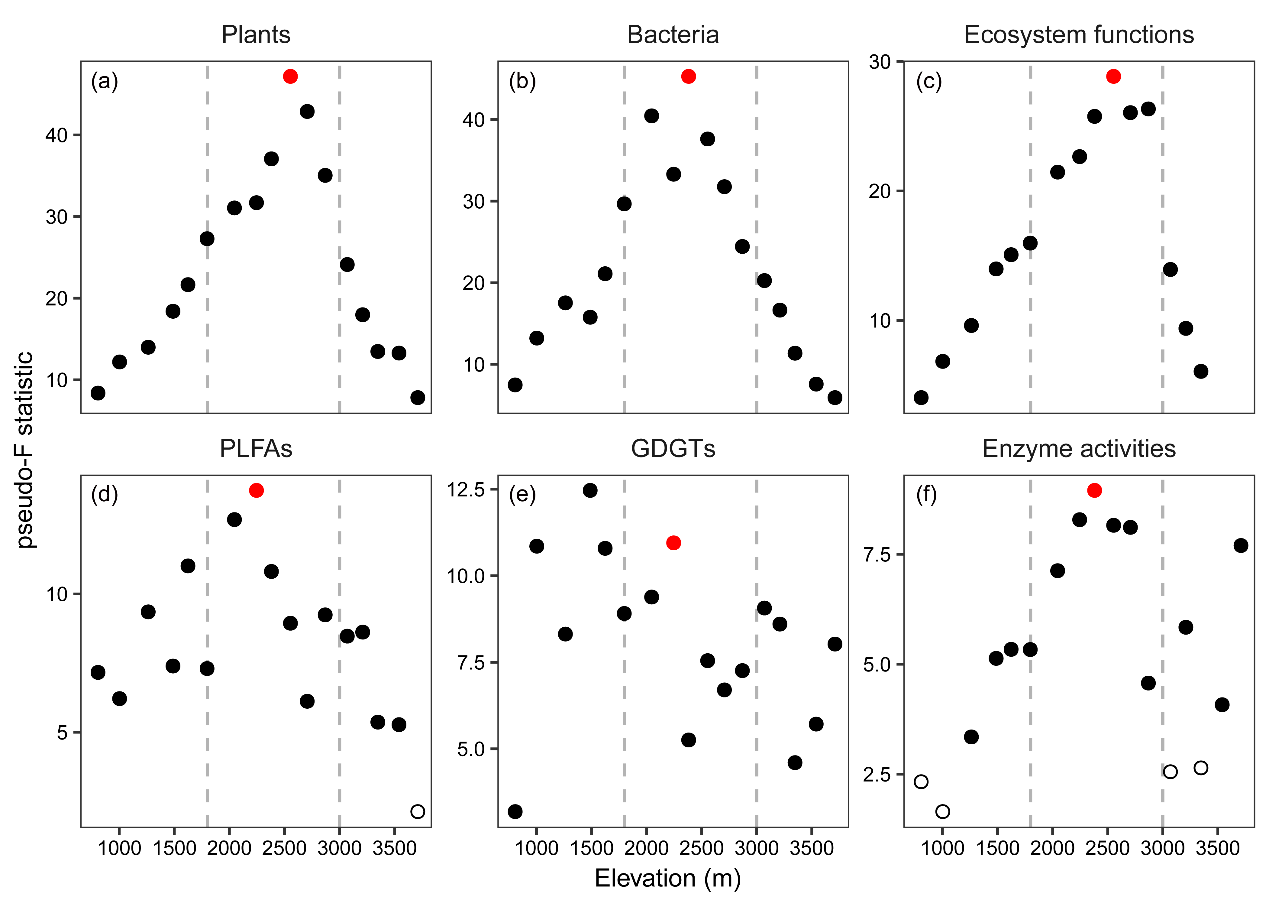


**Figure S5**. Compositional differences of biological communities and ecosystem functions between low and high elevations. For each site, the low and high elevations were defined as the sites below and above its elevation, respectively. The significant differences between the communities of low and high elevations were tested by a permutational multivariate analysis of variance (PERMANOVA) with pseudo-F statistic and were shown as solid and open circles for significance (*P* < 0.05) or non-significance (*P* > 0.05), respectively. Red solid circles indicate the greatest compositional differences occurred within 1800–3000 m elevation range. We considered plant and bacterial communities, and ecosystem functions which include total functions and some functional groups such as phospholipid fatty acids (PLFAs), glycerol dialkyl glycerol tetraether (GDGTs) and enzyme activities.


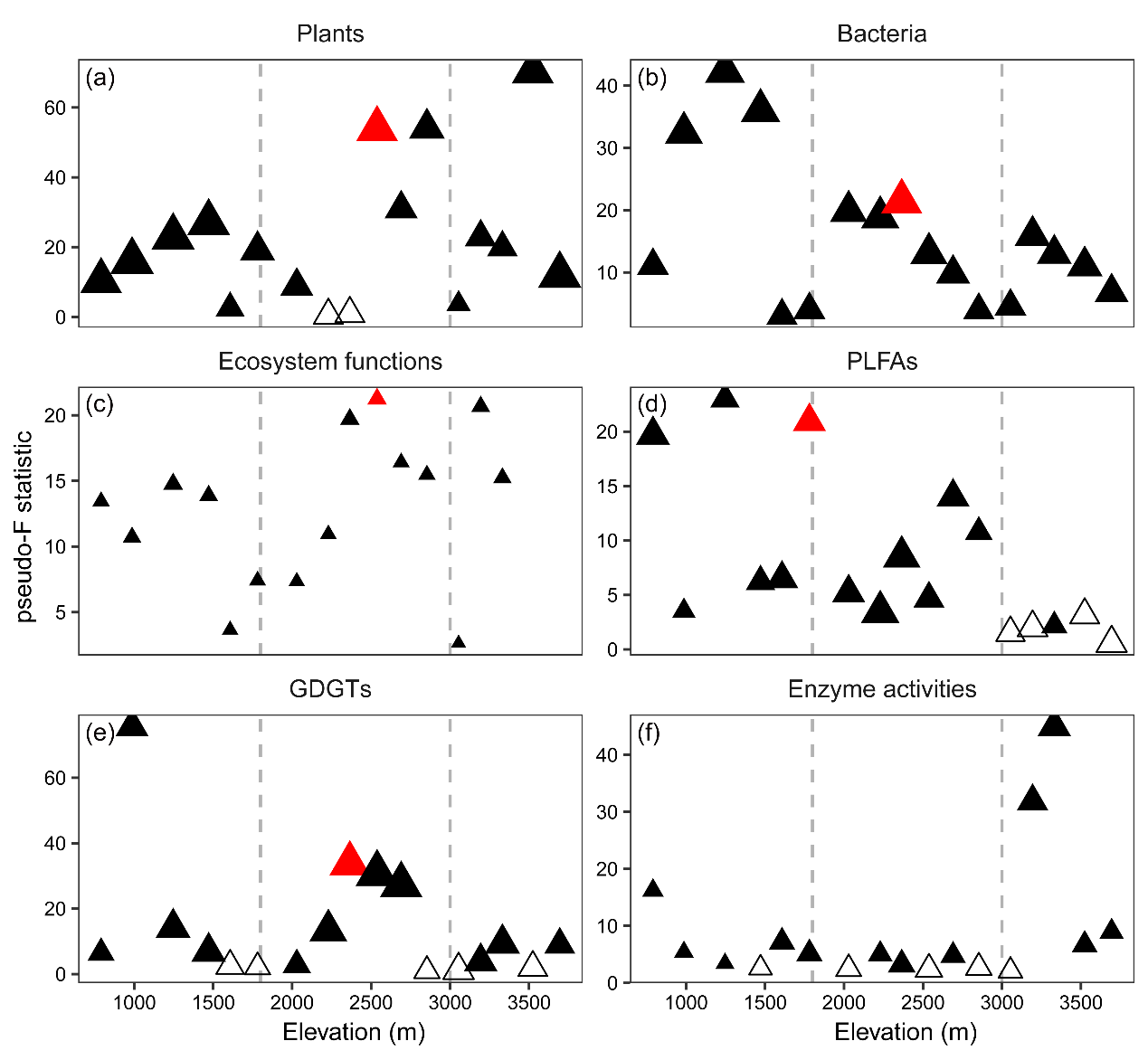


**Figure S6**. The elevational breakpoint estimation of plants, bacteria and ecosystem functions. Elevational variations of Bray-Curtis dissimilarity between adjacent sites were shown for plants (a), bacteria (b), all ecosystem functions (c) and some functional groups including phospholipid fatty acids (PLFAs, d), glycerol dialkyl glycerol tetraether (GDGTs, e) and enzyme activities (f). Triangles show pseudo-F statistic of permutational multivariate analysis of variance (PERMANOVA), which was conducted to evaluate the significance and magnitude of compositional differences between adjacent sites. Solid and open triangles indicate significant (*P* < 0.05) and non-significant (*P* > 0.05) pseudo-F values, respectively, and the sizes of triangles are consistent with dissimilarity values. Red triangle indicates the elevational breakpoint as the highest compositional turnover between adjacent sites within the 1800–3000 m elevation ranges, which has the greatest F-statistic and dissimilarity values.


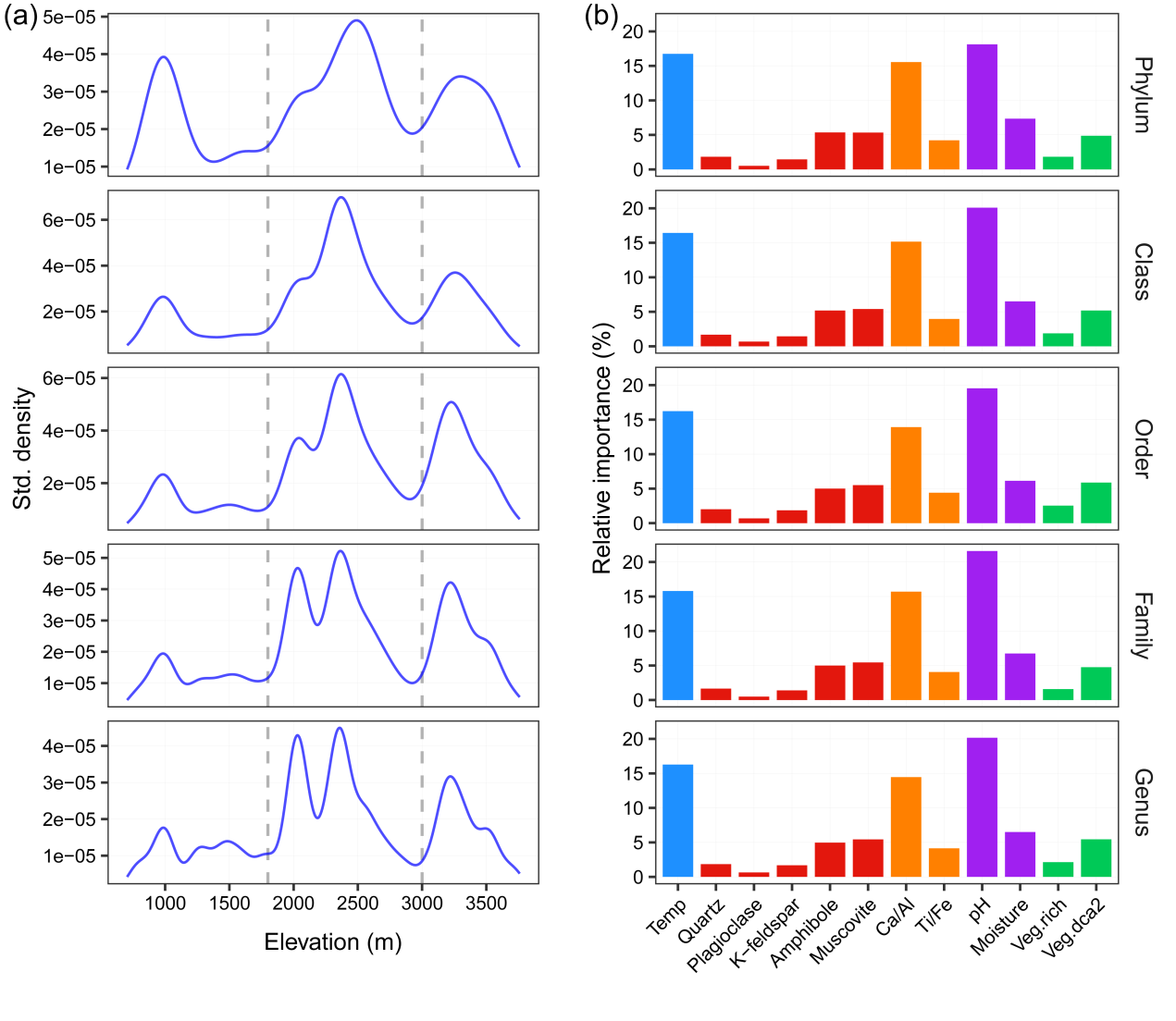


**Figure S7**. The elevational variations and explanatory variables of bacterial composition across taxonomic levels. By using gradient forest analyses, we determined the standardized density of split points on the elevational gradient (a) and the relative influence of explanatory variables (b). We considered the explanatory variables as climate, parent rock, weathering, local and biotic attributes, and the relative importance (%) of each variable are shown as barplots (b). The taxonomic levels include phylum, class, order, family and genus levels. The vertical dashed lines mark the 1800–3000 m elevation ranges. One main peak within 1800–3000 m elevation ranges was more obvious towards higher taxonomic levels, such as phylum, class and order levels.


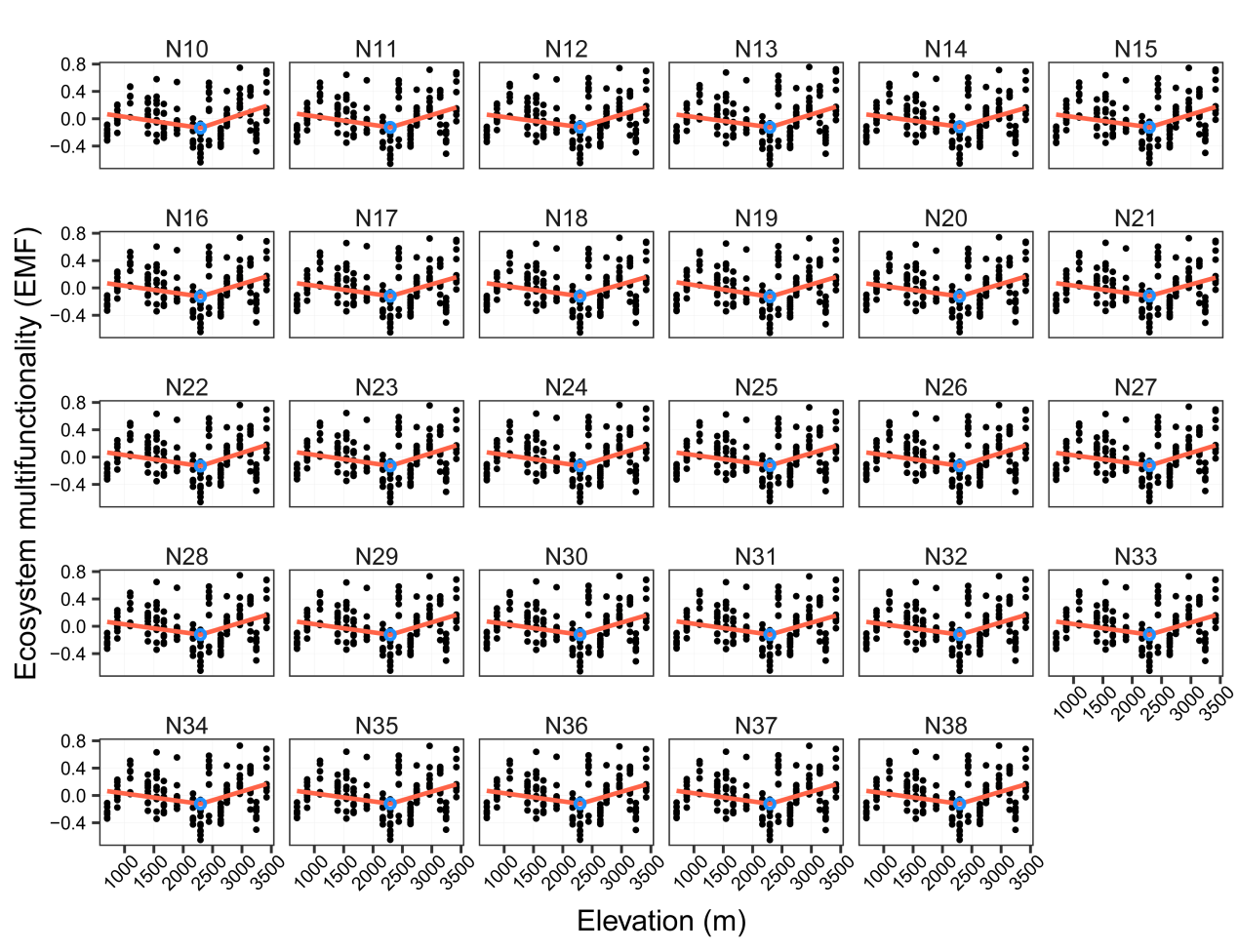


**Figure S8**. The elevational breakpoint estimation for ecosystem multifunctionality (EMF) with the increasing number of ecosystem functions using piecewise regression analyses. EMF was calculated using a series of all possible combinations from 10 to 38 functions with 1000 permutations. The open circles indicate the breakpoint elevations.


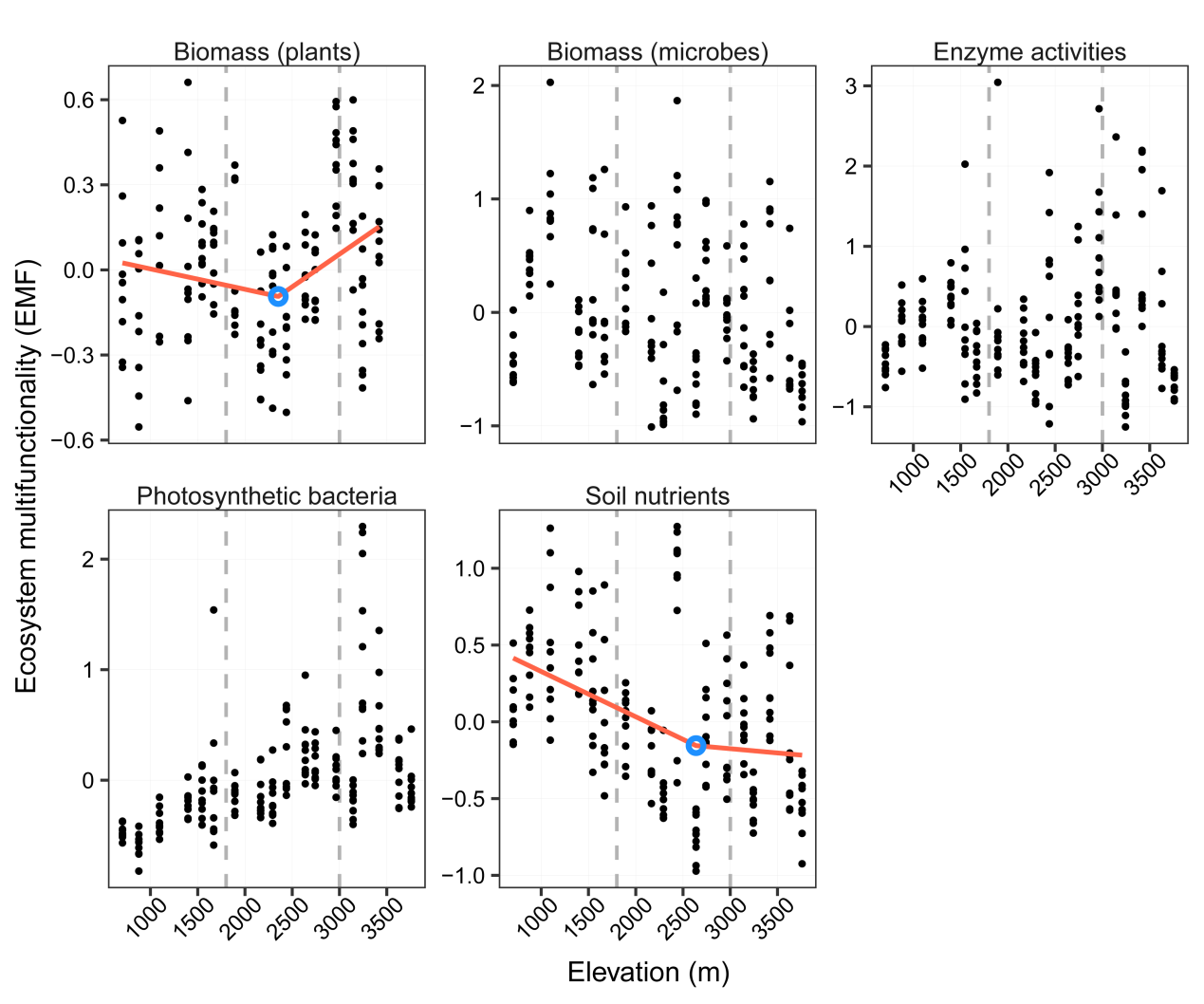


**Figure S9**. The elevational breakpoint estimation of ecosystem multifunctionality (EMF) for functional groups using piecewise regression analyses. EMF was calculated for five functional groups: plant biomass, microbial biomass, enzyme activities, photosynthetic bacteria and soil nutrients. The open circles indicate the breakpoint elevations.


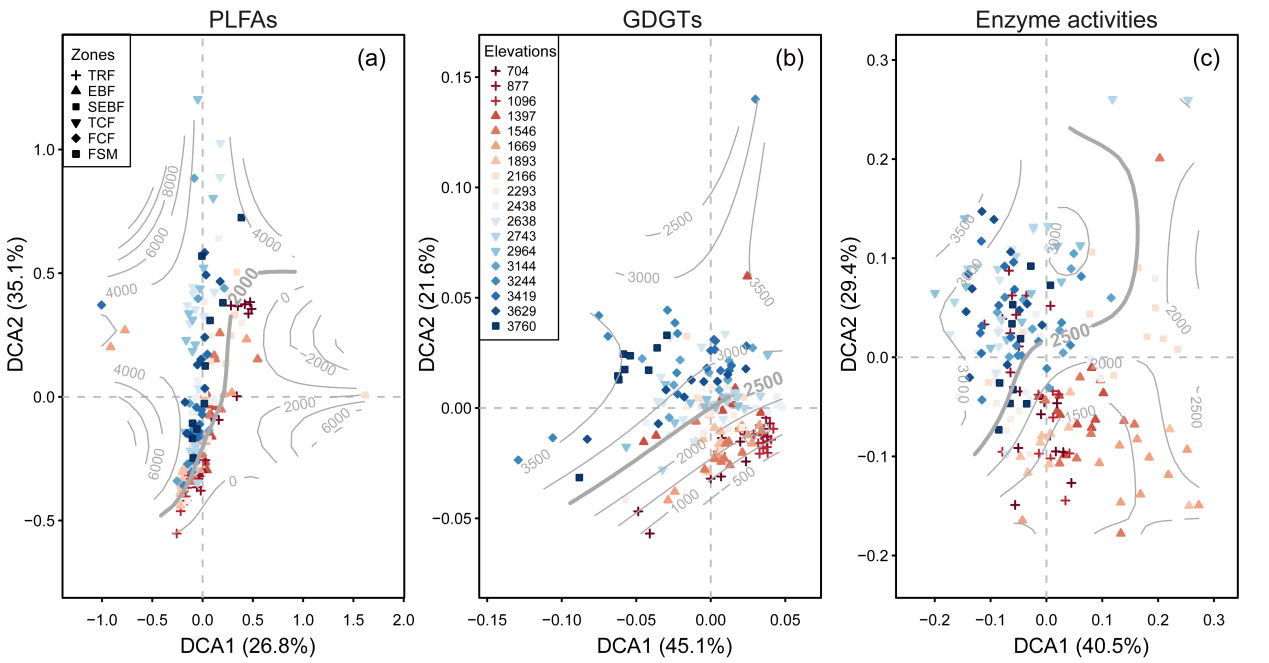


**Figure S10**. Detrended correspondence analyses (DCA) of compositions of ecosystem functional groups. Functional groups include phospholipid fatty acids (PLFAs), glycerol dialkyl glycerol tetraether (GDGTs) and enzyme activities. The contours in grey indicate linear relationships between DCA ordination values and elevations. The compositional differences among pairwise elevations were analyzed to determine the highest compositional turnover as shown with bold lines (Fig. S5). Vegetation zones comprise tropical monsoon rain forest (TRF), subtropical evergreen broadleaved forest (EBF), subtropical evergreen and semi-evergreen broadleaved forest (SEBF), temperate mixed coniferous broadleaved forest (TCF), frigid-temperate coniferous forest (FCF) and frigid shrub meadows (FSM).


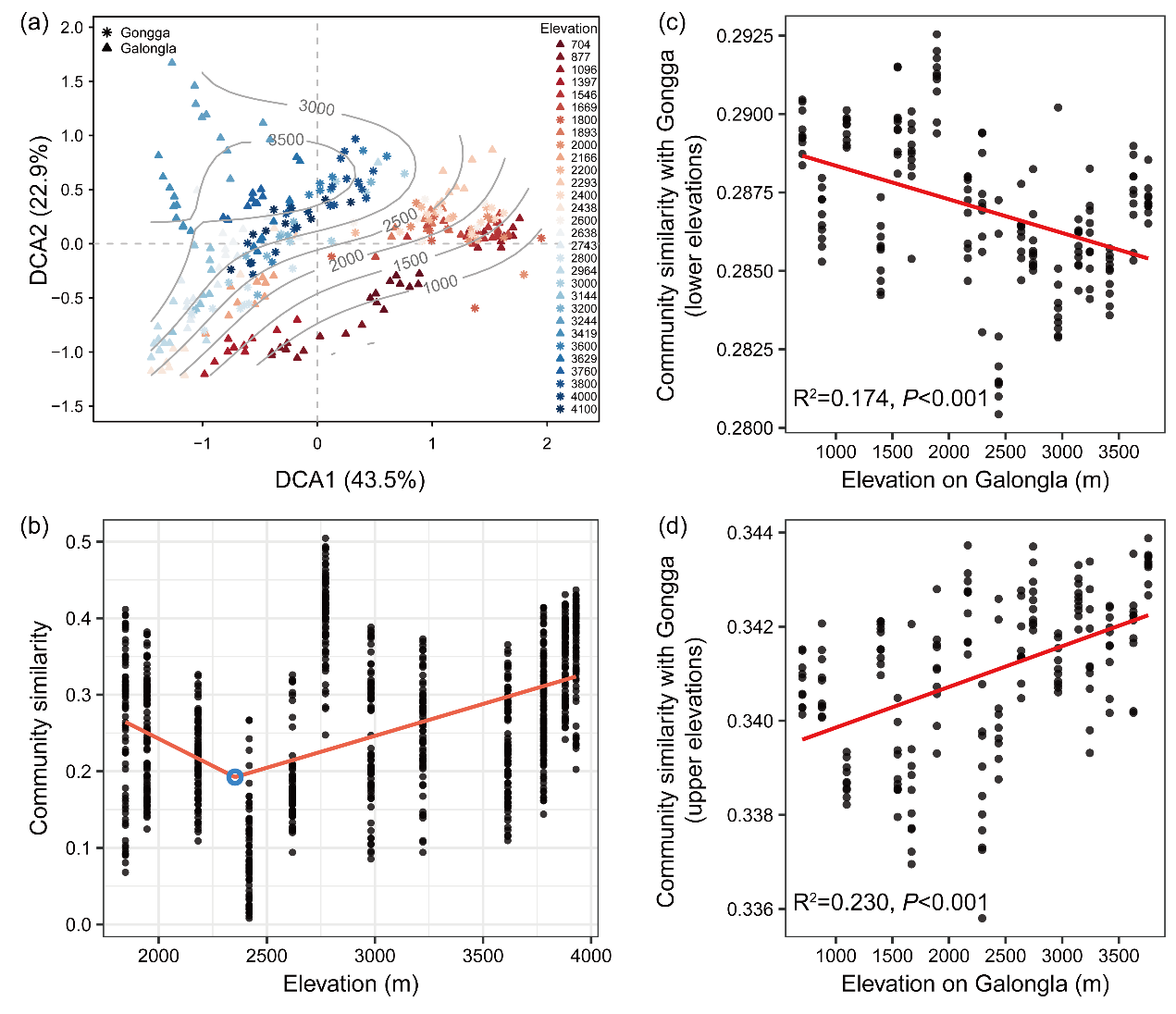


**Figure S11**. Consistent elevational breakpoints of bacterial communities on Galongga Mountain with those reported for Gongga Mountain. (a) Detrended correspondence analysis (DCA) of bacterial community on the two mountains. The contours in grey indicate linear relationships between DCA ordination values and elevations. (b) The elevational variation of bacterial community similarity between pairwise sites with similar elevations of the two mountains. The elevational breakpoint was examined using piecewise regression analyses. The open circle showed the elevational breakpoint at 2360 m. This is supported by the monotonically decreasing (or increasing) patterns for the community similarity between each elevation of Galongla Mountain and all of the lower (or upper) elevations of Gongga Mountain (c, d).


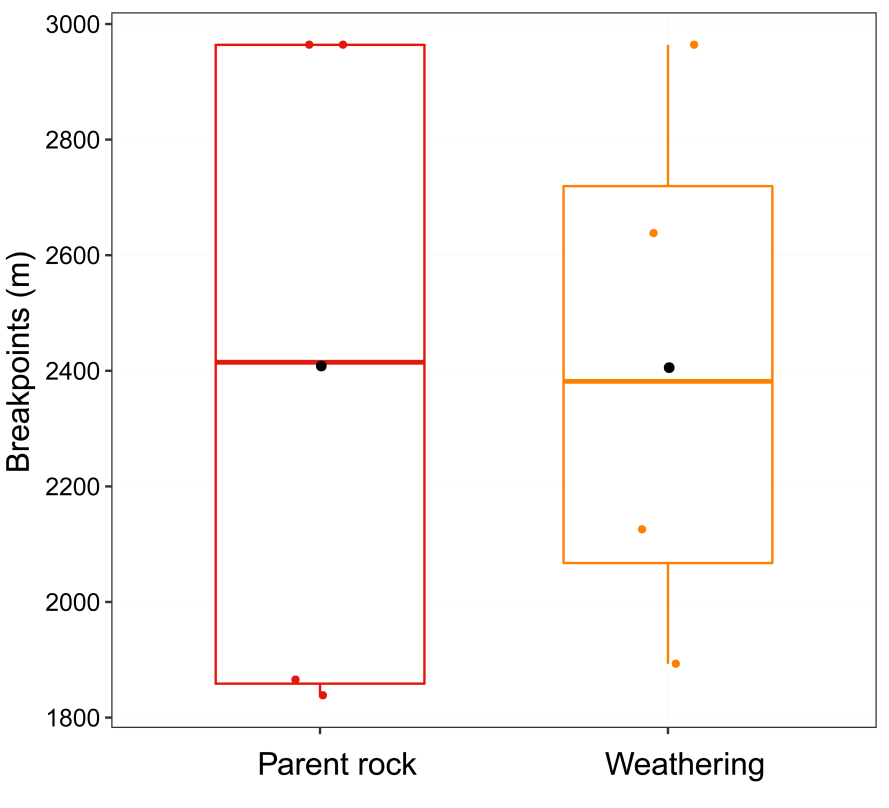


**Figure S12**. The elevational breakpoint estimation for geological variables associated with parent rock and weathering conditions using piecewise regression analyses. Coloured dots in the boxplots are elevational breakpoints for individual explanatory variables, and black dots indicate mean values.


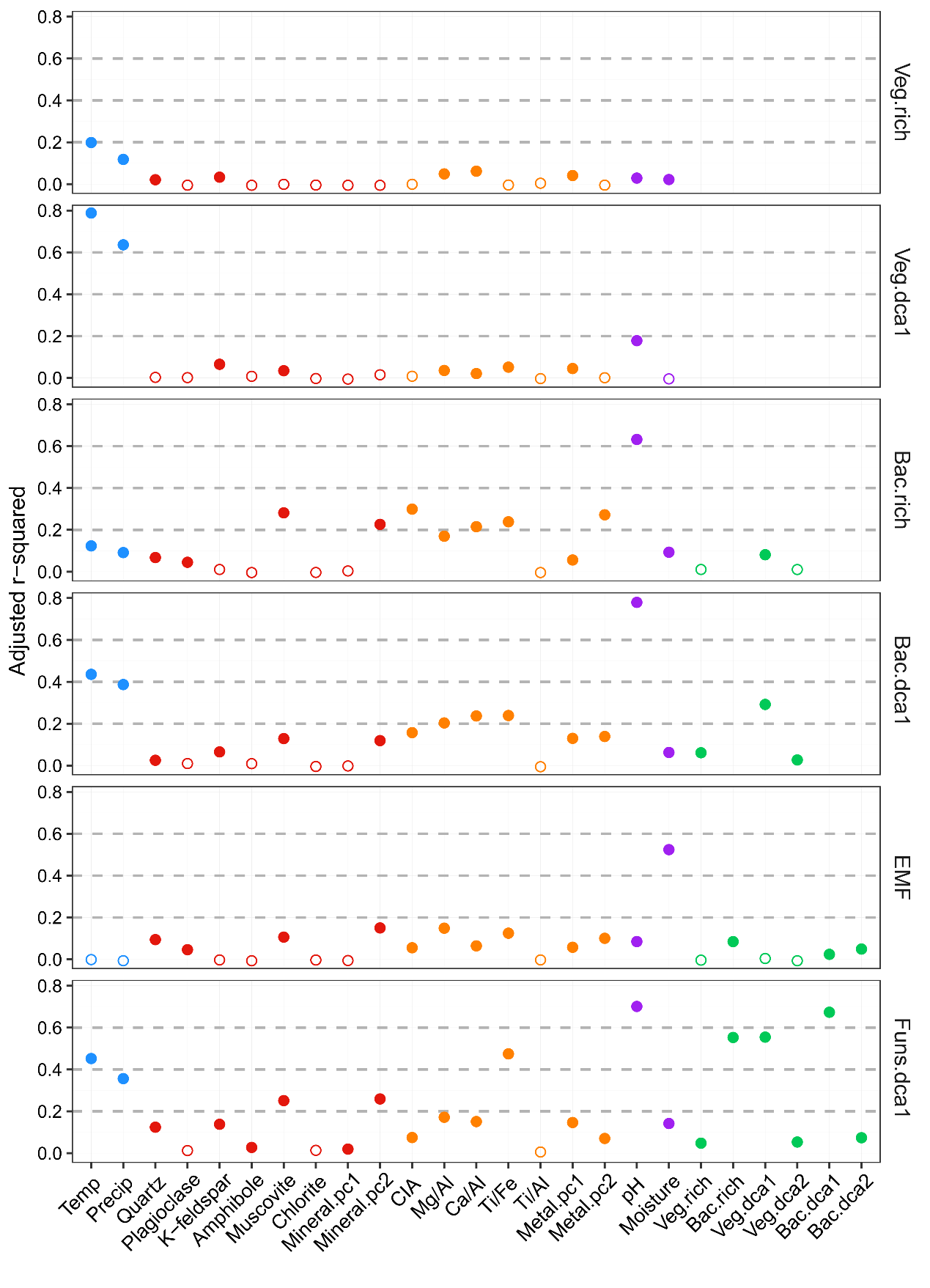


**Figure S13**. The relative influence of explanatory variables on plants, bacteria and ecosystem functions using a linear model based on ordinary least squares regression. For plants and bacteria, we considered diversity with species richness (Veg.rich and Bac.rich) and community composition with the first axis of detrended correspondence analysis (DCA) (Veg.dca1 and Bac.dca1). For ecosystem functions, we considered ecosystem multifunctionality (EMF) and the composition of ecosystem functions with the first axis of DCA (Funs.dca1). Each circle is the adjusted r-squared for individual explanatory variable. Solid and open circles indicate the significant (*P* < 0.05) and non-significant (*P* > 0.05) adjusted r-squared, respectively. The details of abbreviations of explanatory variables are available in Table S3.


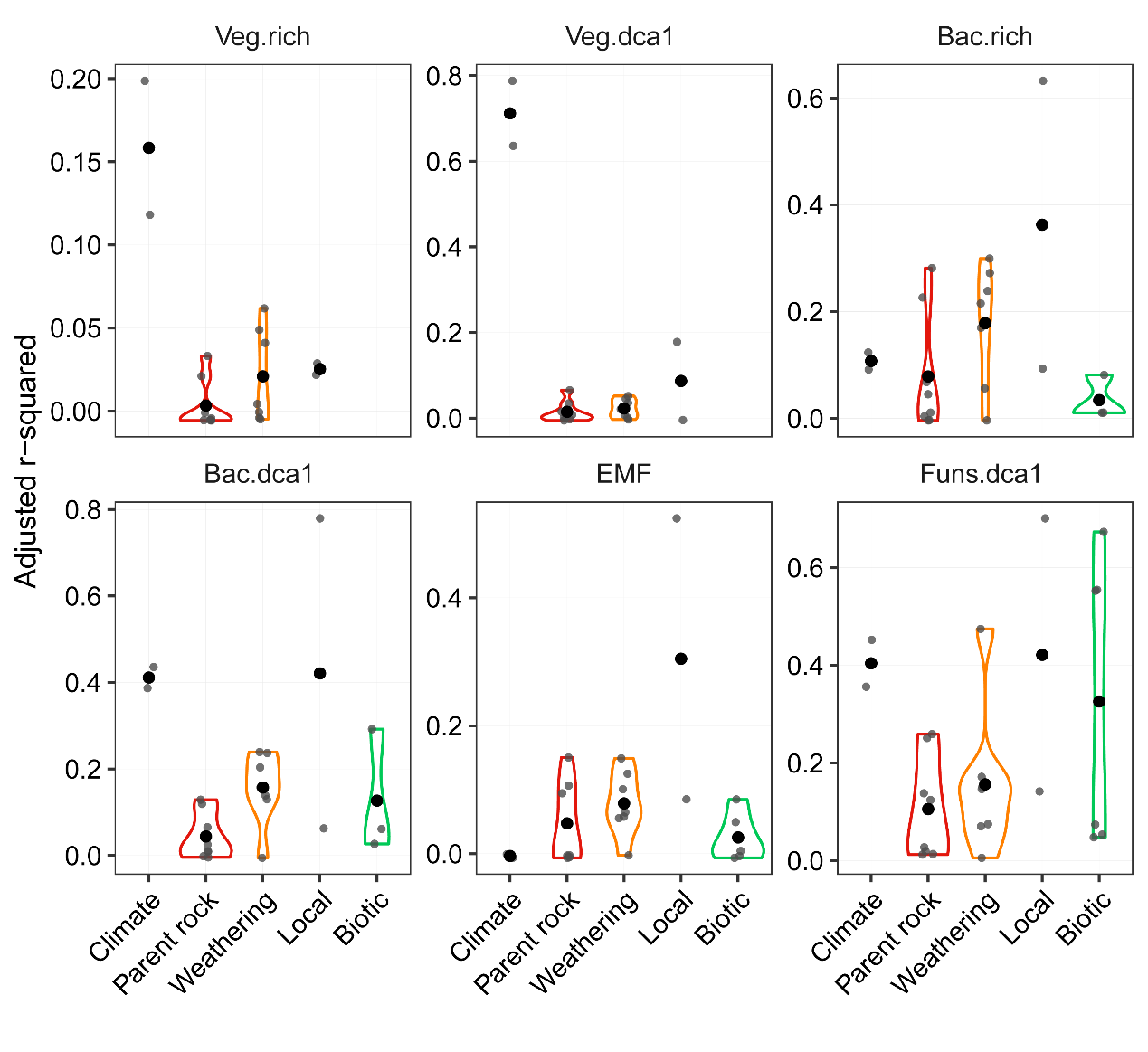


**Figure S14**. Violin plots of the adjusted r-squared according to main driver categories of climate, parent rock, weathering, local and biotic attributes. These adjusted r-squared, based on ordinary least squares regressions, are the same as shown in Fig. S13. Grey dots are the adjusted r-squared for individual explanatory variables, and black dots are the mean values for each category.


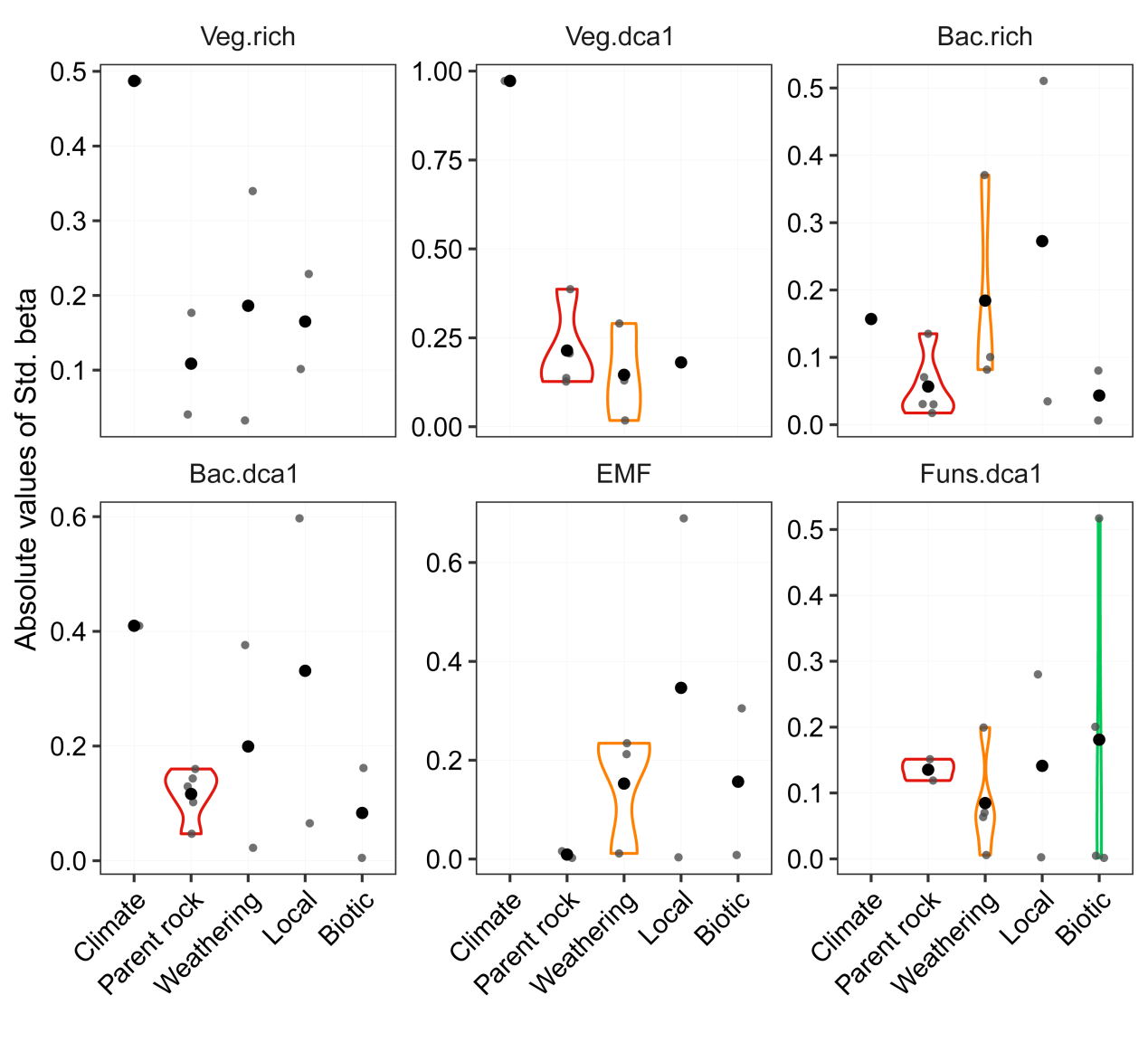


**Figure S15**. Violin plots of the absolute standardized beta weights according to main driver categories of climate, parent rock, weathering, local and biotic attributes. These beta weights were determined by multimodel averaging analyses for plants, bacteria and ecosystem functions, and are the same as shown in Fig. 3a–c. Grey dots are the beta-weights for individual explanatory variables, and black dots are the mean values for each group.


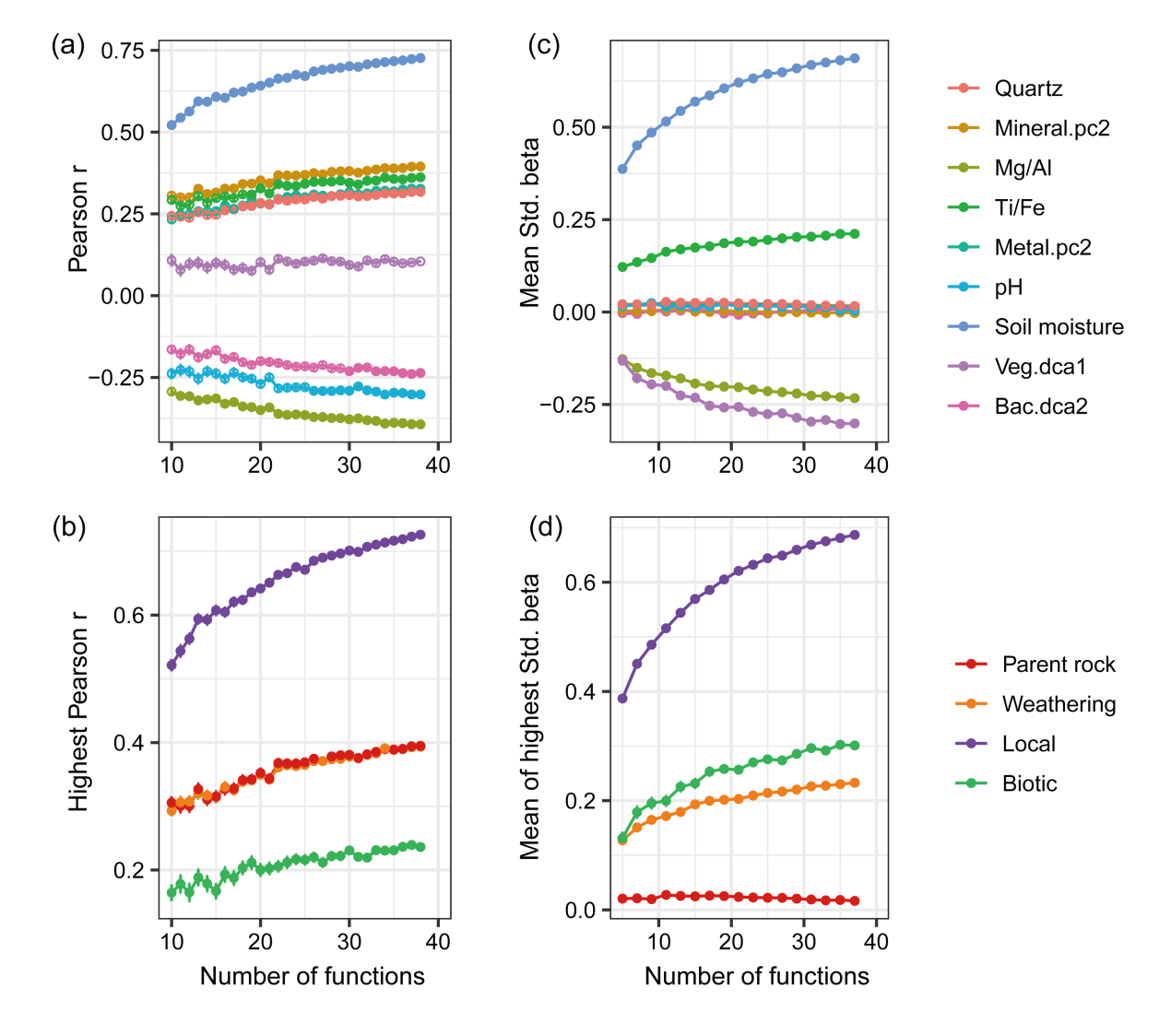


**Figure S16**. The relationships of ecosystem multifunctionality (EMF) and the explanatory variables with the increasing number of ecosystem functions, using multimodel averaging (a–b) and Pearson correlation (c–d) analyses. These analyses were performed to test whether the importance of geological and contemporary processes would increase with the increasing number of functions. Explanatory variables were divided into main driver categories of parent rock, weathering, local and biotic attributes. Totally, we considered nine explanatory variables (a and c) and four groups of explanatory variables (b and d). The relative influence of explanatory variables at the group level was quantified by selecting variables with the highest absolute standardized beta values or Pearson r at parent rock, weathering, local and biotic groups. The details of abbreviations of explanatory variables are available in Table S3. Data are presented as means ± s.e under a random sampling of 1000.


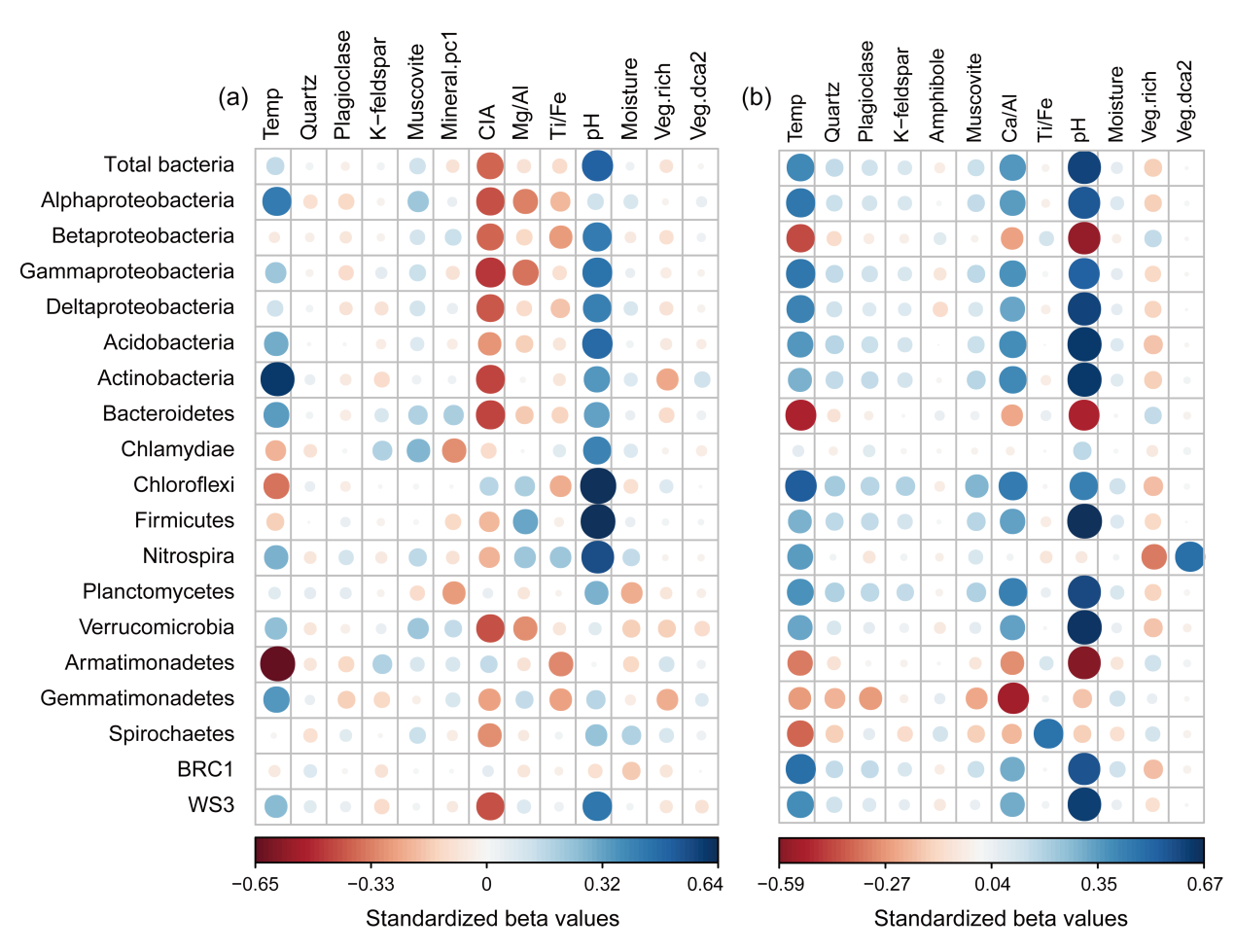


**Figure S17**. The relative influence of contemporary and geological variables explaining the biodiversity (a) and community compositions (b) of bacterial phyla. The biodiversity and community compositions were indicated by species richness and first axis of detrended correspondence analyses (DCA) of the composition of each bacterial phylum, respectively. The dots show the positive (blue) and negative (red) standardized beta weights for each variable using weighted averaging of parameter estimates over best-fitting models in predicting each bacterial phylum based on multimodel averaging analysis. The details of abbreviations of explanatory variables are available in Table S3.


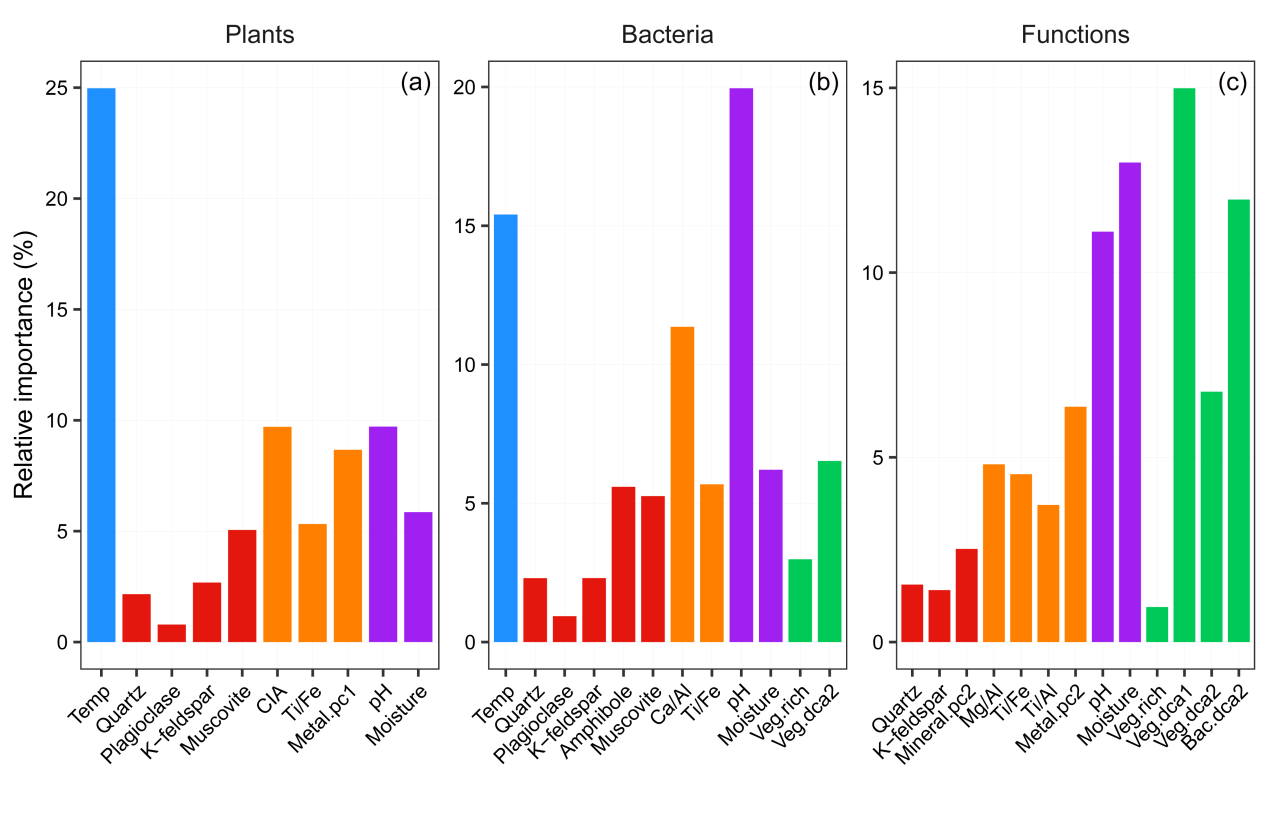


**Figure S18**. Bar plots showing the relative influences of explanatory variables on the composition of plants (a), bacteria (b) and ecosystem functions (c). The relative influences were quantified by gradient forest analyses. Explanatory variables include climate, parent rock, weathering, local and biotic attributes, and the details of abbreviations are available in Table S3.


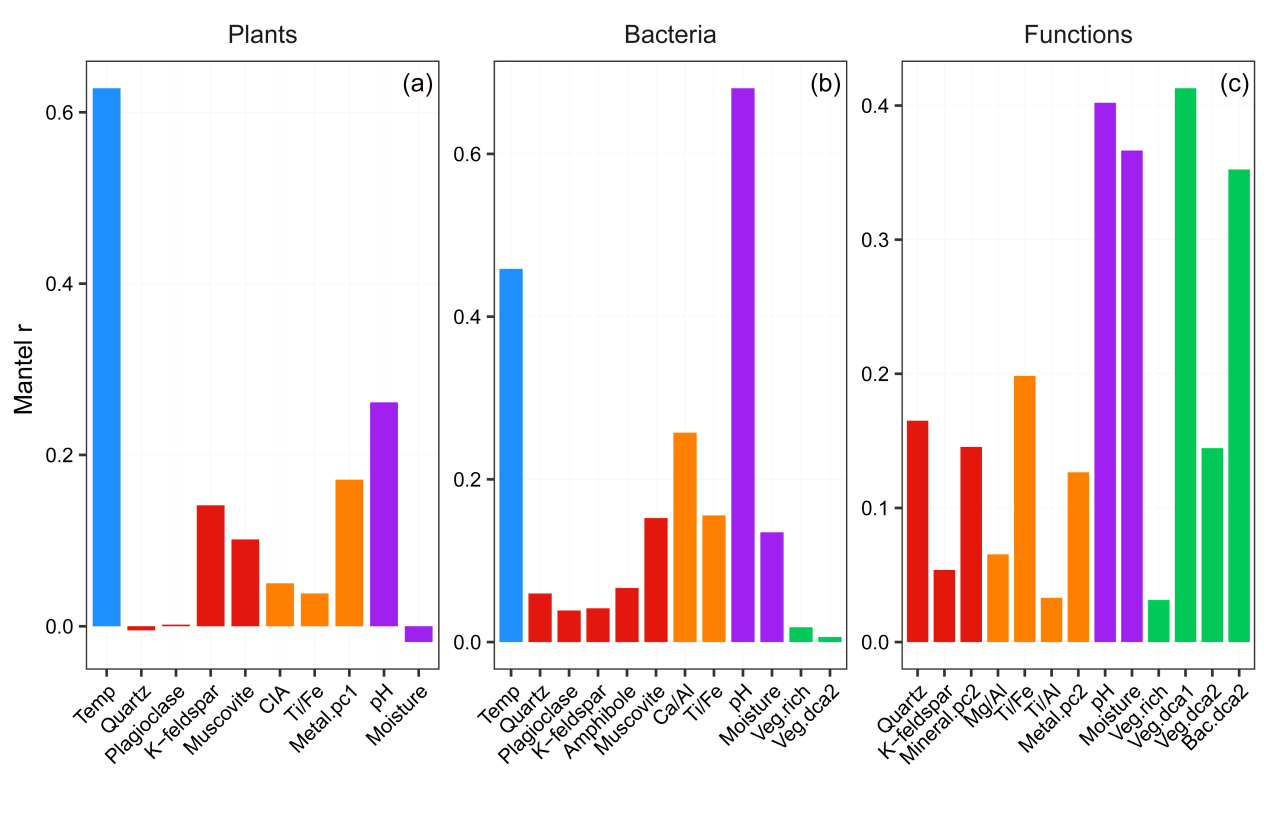


**Figure S19**. Bar plots showing Mantel r between the composition of plants (a), bacteria (b) or ecosystem functions (c) and contemporary or geological variables. The Mantel r was determined by Mantel test with 999 permutations. The details of abbreviations of contemporary and geological variables are available in Table S3.


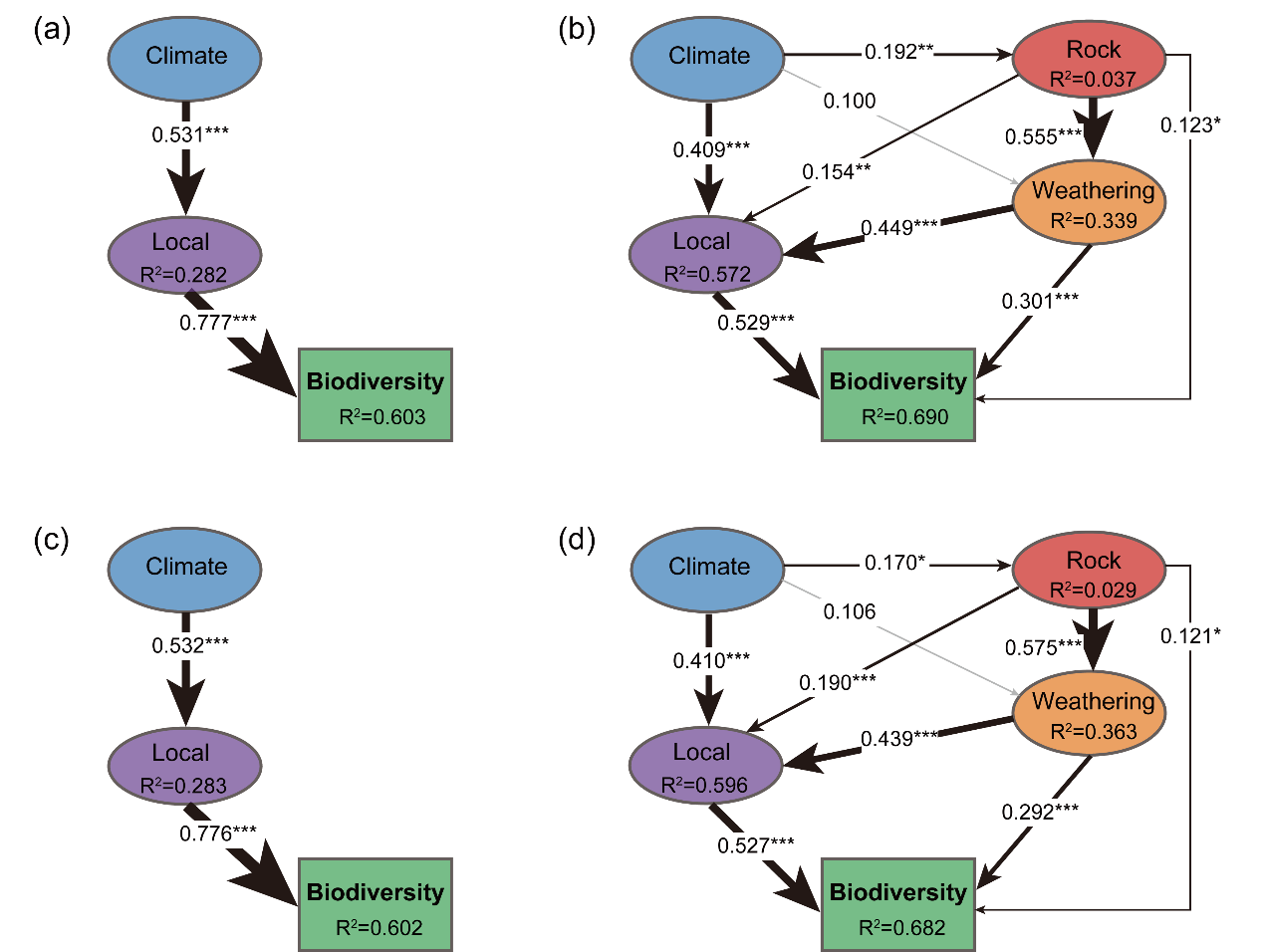


**Figure S20**. Alternative structural equation models (SEM) of multidiversity (MD), which were computed with species richness of bacterial phyla and plants (a–b) or that of bacterial phyla (c–d). Best-fitting models illustrate the effects of predictor variables on MD by excluding (a, c) or including (b, d) geological variables. *R*^2^ denotes the proportion of variance explained for endogenous variables. Grey and black arrows indicate statistically non-significant and significant (****P* < 0.001, ***P* < 0.01, **P* < 0.05) relationships, respectively. Arrow widths and accompanying numbers indicate the relative effects (that is, standardized path coefficients) of modeled relationships. Composite and observed variables are indicated in ovals and rectangles, respectively. More details on model fit summary are provided in Table S8.


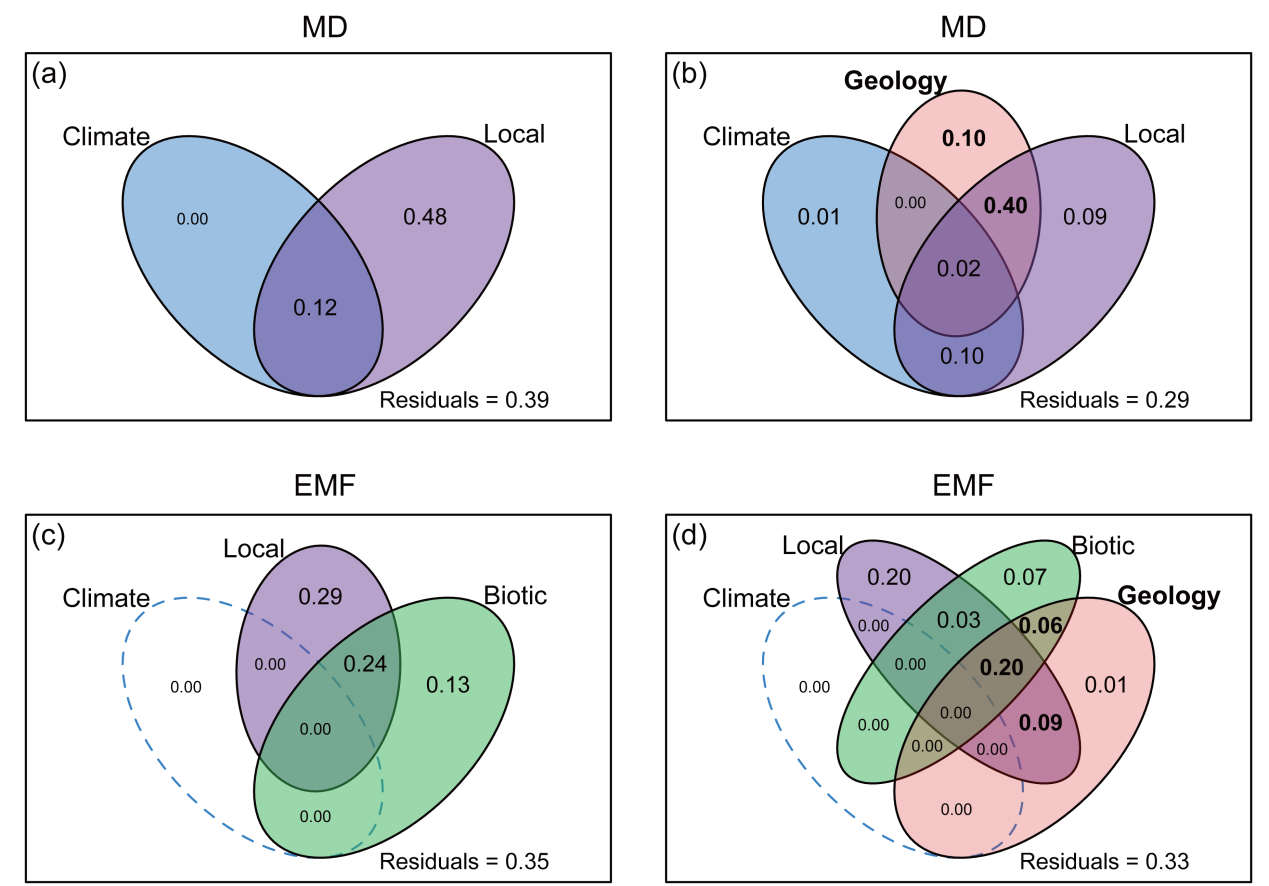


**Figure S21**. The relative influence of explanatory variables on multidiversity (MD, a–b) and ecosystem multifunctionality (EMF, c–d) determined by variation partitioning analysis (VPA). We illustrated the relative influences of explanatory variables on MD (a–b) or EMF (c–d) by excluding (a, c) or including (b, d) geological processes. We considered the explanatory variables as the main driver categories of climate, geology (that is, parent rock, weathering), local and biotic attributes. The unexplained variations are shown as residuals.


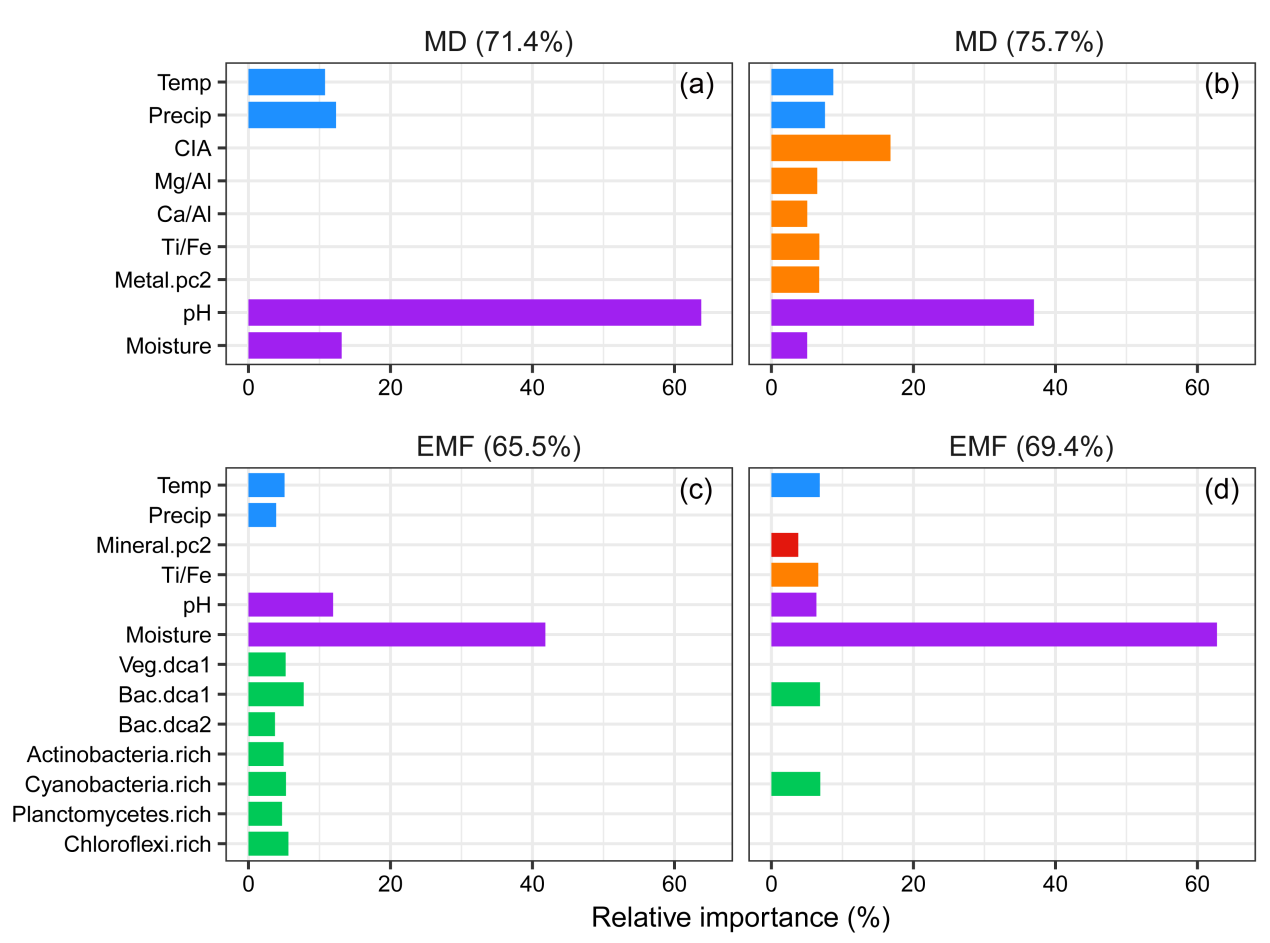


**Figure S22**. The relative influence of explanatory variables on multidiversity (MD, a–b) and ecosystem multifunctionality (EMF, c–d), determined by random forest analyses. We illustrated the relative influences of explanatory variables on MD (a–b) or EMF (c–d) by excluding (a, c) or including (b, d) geological variables, that is, parent rock and weathering. The percentage in parenthesis shows the total explained variances. The relative contribution (%) of each variable for MD and EMF is shown as bar plots. The details of abbreviations of explanatory variables are available in Table S3.
